# Role of oral hyaluronic acid for joint health: insights from rat models and clinical trials

**DOI:** 10.3389/fnut.2025.1691328

**Published:** 2025-12-17

**Authors:** Botao Wang, Fengli Wang, Tianmeng Zhang, Junying Bai, Shumao Cui, Haining Shi

**Affiliations:** 1 Bloomage Biotechnology Co., Ltd., Jinan, China; 2 Key Laboratory of Industrial Biotechnology, Ministry of Education and School of Biotechnology, Jiangnan University, Wuxi, Jiangsu, China; 3 Citrus Research Institute, Southwest University, Chongqing, China; 4 State Key Laboratory of Food Science and Technology, School of Food Science and Technology, Jiangnan University, Wuxi, Jiangsu, China

**Keywords:** hyaluronic acid, osteoarthritis, different molecular weights, clinical trial, nutrition

## Abstract

**Background:**

Early studies have demonstrated the significant potential of hyaluronic acid (HA) in alleviating osteoarthritis (OA); however, the relationship between different molecular weights (MWs) and efficacy remains unclear.

**Methods:**

The rat model was used to evaluate the effects of different MWs of HA on OA and to identify the MW that was most effective in alleviating OA. Based on this, a clinical trial was conducted to verify the selected HA’s clinical efficacy.

**Results:**

The results showed that HA significantly reduced joint swelling in rats, dramatically increased HA content in the serum and joint synovial fluid, decreased serum and joint synovial fluid levels of pro-inflammatory cytokines, and reduced the expression of matrix metalloproteinases (MMPs), inducible nitric oxide synthase (iNOS), and cyclooxygenase-2 (COX-2) when compared with the OA group, especially high-MW HA. Importantly, these protective roles may be attributed to the immune regulation of HA. Clinical trial results indicated that HA significantly decreased pain, stiffness, and physical function of Western Ontario and McMaster Universities Osteoarthritis Index (WOMAC) scores and had no significant impact on blood and urine indices.

**Conclusion:**

Our findings suggest that oral supplementation with HA can reduce the progression of arthritis, pain, and cartilage damage, and can be a new strategy to relieve joint discomfort.

## 1 Introduction

Osteoarthritis (OA) is one of the most common forms of arthritis and a leading cause of global disability (1–3). Current strategies focus on alleviating the symptoms of OA, including Taylor spatial frame, and non-steroidal anti-inflammatory drugs (4, 5), etc. Comprehensive application of high tibial osteotomy, chronic distraction tissue regeneration technology, and computer-assisted external fixation technology showed a protective role in correcting genu varus deformity and recovering the lower limb force line (6). Although these strategies have shown remarkable efficacy in alleviating OA symptoms, many deficiencies and side effects persist, highlighting the urgent need for effective and safe strategies.

Nutritional intervention strategies, which mainly include glucosamine (7), chondroitin sulfate (8), collagen hydrolysates (9), and hyaluronic acid, have attracted much attention because of their great potential for alleviating OA. Among these, hyaluronic acid (HA) is a component of the synovial fluid and plays an important role in the viscoelasticity and lubrication of the knee joint (10). Its molecular weight (MW) varies depending on the length of its molecular polymerization chain. Previously, the intra-articular injection of HA has been used as an alternative non-surgical treatment option for patients with OA (11, 12); however, it requires very high skills and is accompanied by relatively severe pain, which is not conducive to long-term use. Recently, some studies have demonstrated that oral HA can play a role in OA relief (13), and the MW of HA is associated with its role in alleviating OA (13–15). An 8-week randomized, double-blind, placebo-controlled trial found that oral low-MW HA (50–500 kDa) could reduce pain and physical function in patients with OA (15). However, another study found that high-MW HA (650–1,200 kDa) inhibits synovial inflammation and macrophage polarization through the GRP78-NF-κB signaling pathway (16), whereas low-MW HA contributes to cochlear inflammation (17). In conclusion, HA plays a role in affecting OA-related pain and function; however, the relationship between the efficacy of HA with different MWs is unclear.

In the present study, we evaluated the therapeutic effects of HA with different MWs on OA using an animal model and a clinical trial. This includes HA content in the serum, pro-inflammatory cytokines, and the main enzymes involved in the degradation of OA articular cartilage.

## 2 Materials and methods

### 2.1 Animal study

#### 2.1.1 Experimental design

Thirty male Wistar rats (6 weeks old, 150–200 g) were purchased from SPF Biotechnology Co., Ltd. (Beijing, China). All rats were housed in an SPF-grade laboratory animal facility with a temperature (21 °C ± 2 °C) and humidity (55% ± 10%) controlled barrier environment under a standard 12-h light: 12-h dark cycle with free access to water and food. All experiments were approved by the Ethics Committee of Jiangnan University, China [JN.No20220315W0800831(049)].

The rats were divided into five groups (*n* = 6 per group):

To avoid cage effects, the rats were randomly divided into the control group, the model group, and the HA group using a completely random method (lottery method). The control group received an injection of 50 μL saline into the right knee joints. With other groups, 2.0 mg of monosodium iodoacetate (MIA) (Sigma, St. Louis, MO, USA) was dissolved in 50 μL saline (40 mg/mL) and injected into the right knee joints through the infrapatellar ligament using a 0.1 mL insulin syringe fitted with a 30-G needle (18–20). Besides, 21 mg of HA was dissolved in 10 mL of ddH_2_O (2.1 mg/mL). The details of the intervention are as follows:

1. Control group: Gavaged with 2 mL day^−1^ PBS for 4 weeks.
2. Model group: Gavaged with 2 mL day^−1^ PBS for 4 weeks.
3. HA1 group: Gavaged with HA (2000 Da, 21 mg/kg day^−1^ BW) for 4 weeks.
4. HA2 group: Gavaged with HA (500–700 kDa, 21 mg/kg day^−1^ BW) for 4 weeks.
5. Ultra HA-J group: Gavaged with HA (>800 kDa, 21 mg/kg day^−1^ BW) for 4 weeks.

After intervention, all rats were anesthetized with 3% isoflurane and sacrificed by decapitation. The animal carcasses were handed over to the solid waste disposal company for processing.

#### 2.1.2 Measurement of joint swelling

Knee joint swelling was assessed in all rats. The assessment consisted of the evaluation of pain and inflammation by measuring the joint diameter. Right knee joint thickness measurements were obtained using an electronic digital caliper and repeated three times. The results were expressed as an average in millimeters.

#### 2.1.3 Measurement of the joint Mankin score

The extent of articular cartilage damage in each joint was evaluated according to the Mankin scoring system (Table 1) by an experienced senior surgeon blinded to the study groups.

**Table 1:** Cartilage evaluation according to the Mankin score system.

Criteria	Score	Histological finding
Structure	0	Smooth intact surface
	1	Slight surface irregularities
	2	Pannus/surface fibrillation
	3	Clefts into the transitional zone
	4	Clefts into the radial zone
	5	Clefts into the calcified zone
	6	Total disorganization
Cells	0	Uniform cell distribution
	1	Diffuse cell proliferation
	2	Cell clustering
	3	Cell loss
Safranin O-Fast Green	0	Normal
	1	Slight reduction
	2	Moderate reduction
	3	Severe reduction
	4	No dye noted
Tidemark integrity	0	Intact
	1	Vascularity

#### 2.1.4 Serum analysis

Blood samples were incubated at room temperature for 2 h and centrifuged at 3000 × *g* for 15 min. Supernatants were collected to determine serum-related indicators. The serum levels of TNF-α, IL-1β, and HA were determined using commercial ELISA kits (TNF-α: cat no. E-EL-R2856c, Elabscience Biotechnology Co., Ltd., Wuhan, China; IL-1β: cat no. E-EL-R0012c; Elabscience Biotechnology Co., Ltd., Wuhan, China; Quantikine Hyaluronan ELISA Kit; R&D Systems, Minneapolis, MN, USA).

#### 2.1.5 Joint synovial fluid analysis

A 30-gauge insulin syringe was directed behind the patella and along the femoral groove. Sterile saline (200 μL) was injected, the needle removed, and the knee was fully flexed 10 times. A clean 30-gauge insulin syringe was then placed along the same path, and back pressure was used to collect as much fluid as possible. The collected joint lavage fluid was centrifuged at 300 × *g* and 4 °C for 5 min. Subsequently, 100 μL of the supernatant was collected and stored in a − 80 °C freezer for subsequent biomarker analysis. Notably, although we did not standardize the collection and extraction of synovial fluid, the data (including HA, cytokines, NO, and PGE2 levels) obtained are based on the relative content of different groups rather than absolute quantification. The changes in its relative content can reflect the activity level of the body’s immune response and the progression of the disease. The cell pellet from the joint lavage fluid was resuspended in flow cytometry buffer for subsequent analysis.

#### 2.1.6 Gene expression analysis

Total RNA was extracted from the articular cartilage tissues using Vazyme and purified using the Fast Pure Cell/Tissue Total RNA Isolation Kit (Vazyme Biotech Co., Ltd., Nanjing, China), according to the manufacturer’s protocol. Total RNA was reverse transcribed into complementary DNA (cDNA) using HiScript III All-in-One RT SuperMix Perfect for qPCR (Vazyme Biotech Co., Ltd., Nanjing, China). The mRNA expression levels of *GAPDH*, *MMP9*, *iNOS*, *COX2*, *MMP3*, and *MMP13* were detected. Primer sequences are listed in Table 2. Relative fold changes in target gene expression between groups were determined for all targets using the 2^∆∆Ct^ method (21, 22).

**Table 2:** Primers for real-time polymerase chain reaction (PCR) analysis of gene expression

Gene	Forward primer (5′ → 3′)	Reverse primer (5′ → 3′)
MMP9	CCCAACCTTTACCAGCTACTC	GTCAGAACCGACCCTACAAAG
iNOS	CTTTACGCCACTAACAGTGGCA	AGTCATGCTTCCCATCGCTC
COX2	CCTCGTCCAGAIGCTATCTTG	GAAGGTCGTAGGTTTCCAGTATT
MMP3	CAGTCCTGCTGTGGCTGTGTAC	AACCTCCATGCCAGCATCTTCTTC
MMP13	AACCAAGATGTGGAGTGCCTGATG	CACATCAGACCAGACCTTGAAGGC

#### 2.1.7 Histological analysis

The rats were sacrificed, and the knee joints were dissected and fixed in 4% polyformaldehyde for 24 h at room temperature, decalcified with 5% formic acid for 4 weeks, dehydrated in graded acetone, and embedded in paraffin. Sections (thickness, 4–5 μm) were stained with hematoxylin and eosin (H&E) and Safranin-O/Fast green to evaluate structural cartilage damage and the proteoglycan loss, respectively, and then observed under a Carl Zeiss Axio Imager A2 microscope (Carl Zeiss, Deisenhofen, Germany). All stained slides were histologically evaluated and statistically graded on a scale of 0–14 using double-blind observation according to the modified Mankin scoring system.

#### 2.1.8 Analysis of CD45+ cells in synovial fluid

CD45+ cells were analyzed using flow cytometry (Aria III; Becton, Dickinson & Co., Sparks, MD, USA) using antibodies against CD45 antibody (Biolegend, San Diego, CA, USA, Cat. # 202216), CD3 antibody (Biolegend Cat. # 201403), CD45RA (Biolegend Cat. # 202313), CD43 (Biolegend Cat. # 202816), CD11b/c (Biolegend Cat. # 201819), and CD86 antibody (Biolegend Cat. # 200307) according to the manufacturer’s Cell populations and flow cytometry data were analyzed using a FACSCalibur flow cytometer (BD Biosciences, San Jose, CA, USA) and FlowJo software (Tree Star, Ashland, OR, USA), respectively.

### 2.2 Clinical trial

#### 2.2.1 Study design

This pilot, randomized, double-blind, placebo-controlled clinical trial was performed. Healthy Japanese people (persons with a Kellgren-Lawrence classification of 0 or 1 on screening and ingestion radiography) were screened for enrolment. The study complied with the ethical guidelines of the Declaration of Helsinki (clinicaltrials.gov, NCT05683327), and its protocol was approved by the Medical Corporation Seishin Kai Takara Clinic Ethics Committee (approval number: 2212-06793-0003-0E-TC). All the participants provided written informed consent for participation. The trial was performed according to the CONSORT guidelines.

Two visits have been scheduled, starting from preliminary screening, followed after 7–14 days by an enrollment visit, when patients were randomized to an indistinguishable placebo or active treatment (UltraHA J, sodium hyaluronate, characterized by a large spectrum of MW, kindly furnished by Bloomage Biotechnology Corp. Ltd., Jinan, China) at 150 and 80 mg/day, respectively. The enrolled patients visited the hospital after 6 (T42) and 12 (T84) weeks.

#### 2.2.2 Criteria for eligibility of patients

The patient inclusion criteria were as follows: (1) Healthy Japanese adults; (2) 23 kg/m^2^ ≤ body mass index (BMI) < 30 kg/m^2^; (3) Subjects who are judged in the Kellgren-Lawrence grade (KL grade) as either 0 or 1 in X-ray; (4) Subjects whose McMaster Universities Osteoarthritis Index (WOMAC) score is relatively high.

The exclusion criteria were as follows: Subjects (1) who are undergoing medical treatment or have a medical history of malignant tumor, heart failure, and myocardial infarction; (2) have a pacemaker or an implantable cardioverter defibrillator; (3) currently undergoing treatment for any of the following chronic diseases: cardiac arrhythmia, liver disorder, kidney disorder, cerebrovascular disorder, rheumatism, diabetes mellitus, dyslipidemia, hypertension, or any other chronic diseases; (4) who use or take “Foods for Specified Health Uses,” or “Foods with Functional Claims” in daily; (5) who consciously consume foods that contribute to knee joint improvements, such as collagen and chondroitin sulfate; (6) who are currently taking medications (including herbal medicines) and supplements; (7) who are allergic to medications and/or the test-food-related products (particularly alcohol); (8) who are pregnant, lactating, or planning to become pregnant during this trial; (9) who suffer from COVID-19; (10) who have been enrolled in other clinical trials within the last 28 days before the agreement to participate in this trial or plan to participate another trial during this trial; (11) who are judged as ineligible to participate in this study by the physician.

#### 2.2.3 Treatment

All participants were randomized and divided in a ratio of 1:1:1 and stratified according to sex and age as follows: HA-80 group (80 mg day^−1^ Ultra HA-J for 12 weeks), HA-150 group (150 mg day^−1^ Ultra HA-J for 12 weeks), and placebo group (0 mg day^−1^ Ultra HA-J for 12 weeks). All test foods possessed identical excipient compositions and were encapsulated in capsule form. The samples in the placebo group were devoid of HA, and their weights were supplemented with microcrystalline cellulose (Table 3). An alphabetical code was assigned to each lot (corresponding to the treatment or placebo) and impressed on the dosing box. The study staff and the investigators, as well as all of the volunteers, were blinded to the group assignment. The codes were stored in a sealed envelope that was not opened until the end of the trial. Dose boxes were mixed, and a blinded dose box was assigned to each enrolled patient.

**Table 3:** Composition of test food.

Group	UltraHA J (mg)	Crystalline cellulose (mg)	Silica (mg)	Calcium stearate (mg)
HA-80	40	116.8	1.6	1.6
HA-150	75	81.8	1.6	1.6
Placebo	0	156.8	1.6	1.6

Treatment compliance was assessed by counting the number of pills returned during specified clinic visits. All unused pills were retrieved from the inventory. All the treatment products were provided free of charge. All participants completed the following points in compliance: (1) Consume the test food according to the specified usage and dosage; (2) Intake of test foods to 80% or more; (3) Avoid overeating and do not change their lifestyle from the date of obtaining the test consent form until the final test (test 12 weeks after intake); (4) Avoid eating as many foods as possible that contribute to knee joint improvement, such as collagen and chondroitin sulfate; (5) Drinking and excessive exercise are not carried out from the day before inspection to the end of the inspection on the day; (6) Eating and drinking are prohibited 6 h before blood collection. Consumption of test foods was also prohibited. However, only water was permitted. Functional drinking of water and tea is not allowed. (7) If a change in physical condition occurs during the examination period, the accredited clinical trial body is immediately contacted, and the following instructions are provided; (8) During the test period, do not consume food or beverages that can be used for specific health food, functional labeling food, and other functions. (9) During the test period, infection prevention measures such as coronavirus and the like (thorough hand washing, hand disinfection, mask-wearing, etc.) are thorough, and if there is suspicion of infection, contact the accredited clinical testing agency promptly.

#### 2.2.4 Outcome assessment

Participants were assessed by screening, pre-ingestion (T1), and 12 weeks after ingestion (T2). There were 24 questions, 5 for pain, 2 for stiffness, and 17 for physical function, with responses ranging from 0 (least severe) to 4 (most severe) on a 96 WOMAC score.

A visual analog scale (VAS) was administered to the enrolled patients at T1 and T2. There is a line of 100 mm at both ends showing the degree of the best and worst, and the degree of the current feeling is marked on the line, where the best state imaginable is 0 and the worst is 100. Clinical evaluation included a physical examination of the affected knee. Adherence, tolerability, and acceptability of the tested treatments were also assessed at T1 and T2. Western Ontario and McMaster Universities Osteoarthritis Index (WOMAC), Visual Analogue Scale (VAS), Kellgren-Lawrence classification, and the blood indicators, etc., were included as primary endpoints in this study.

#### 2.2.5 Safety evaluation

In clinical trials, the blood indicators (white blood cell count, red blood cell count, hemoglobin, hematocrit, platelet count, aspartate aminotransferase, alanine aminotransferase, gamma-glutamyl transpeptidase, total bilirubin, total protein, urea nitrogen, creatinine, uric acid, sodium, potassium, chloride, serum amylase, total cholesterol, HDL-cholesterol, LDL-cholesterol, triglycerides, glucose, and hemoglobin A1c) and urine indicators (urine protein, urine glucose, urine pH, and urine occult blood) were detected to evaluate the security of HA.

### 2.3 Statistical analysis

Experimental data analysis in the animal experiment section: The data are presented as means ± standard deviations (SD), and the Shapiro–Wilk test is used to verify the normality of the data. For data that follow a normal distribution, non-paired *t*-test, one-way analysis of variance (ANOVA), and Dunnett’s multiple comparison test are used to analyze the significance of differences; for non-normal distribution data, the Mann–Whitney non-parametric test, Kruskal-Wallis non-parametric test, and Dunn’s *post-hoc* test are used to analyze significance; the significance criterion is set at *p* < 0.05. With clinical trial, mean difference (MD) and its 95% confidence intervals (CIs) were used for calculating the pooled data. Group comparisons were conducted using Analysis of Covariance (ANCOVA). This ANCOVA showed the mean values and standard deviations, with baseline (before screening and intake) as the covariate. All statistical analyses were performed using GraphPad Prism 6 (version 6.01; GraphPad Software Inc., San Diego, CA, USA).

## 3 Results

### 3.1 HA suppressed joint diameter in MIA-induced OA rats

**Figure 1:**
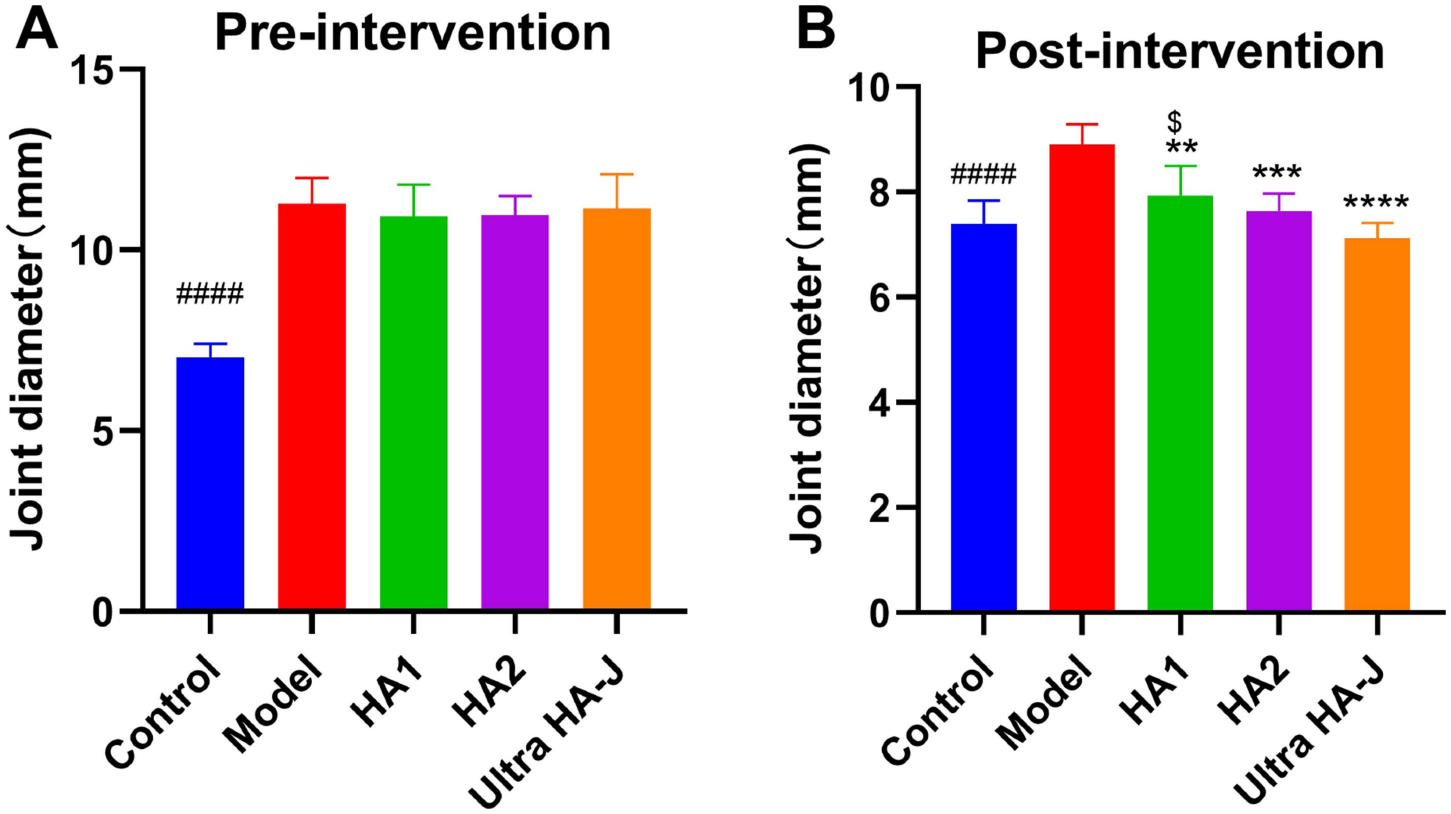
Joint diameter during pre-intervention **(A)** and post-intervention **(B)** of MIA-induced OA rats. #### *p* < 0.0001, control group vs. model group; **p < 0.01, ***p < 0.001, ****p < 0.0001, intervention group vs. model group; $*p* < 0.05, HA1 group vs. ultra-HA-J group.

Joint diameter is the main index used to assess knee swelling. As shown in Figure 1A, MIA significantly enhanced the joint diameter compared to the control group, suggesting successful construction of the OA model. After the intervention, compared to the model group, all forms of HA dramatically reduced the joint diameter. Notably, the inhibitory effect on the joint diameter became more pronounced with increasing HA molecular weight (Figure 1B).

### 3.2 HA suppressed articular cartilage damage in MIA-induced OA rats

**Figure 2:**
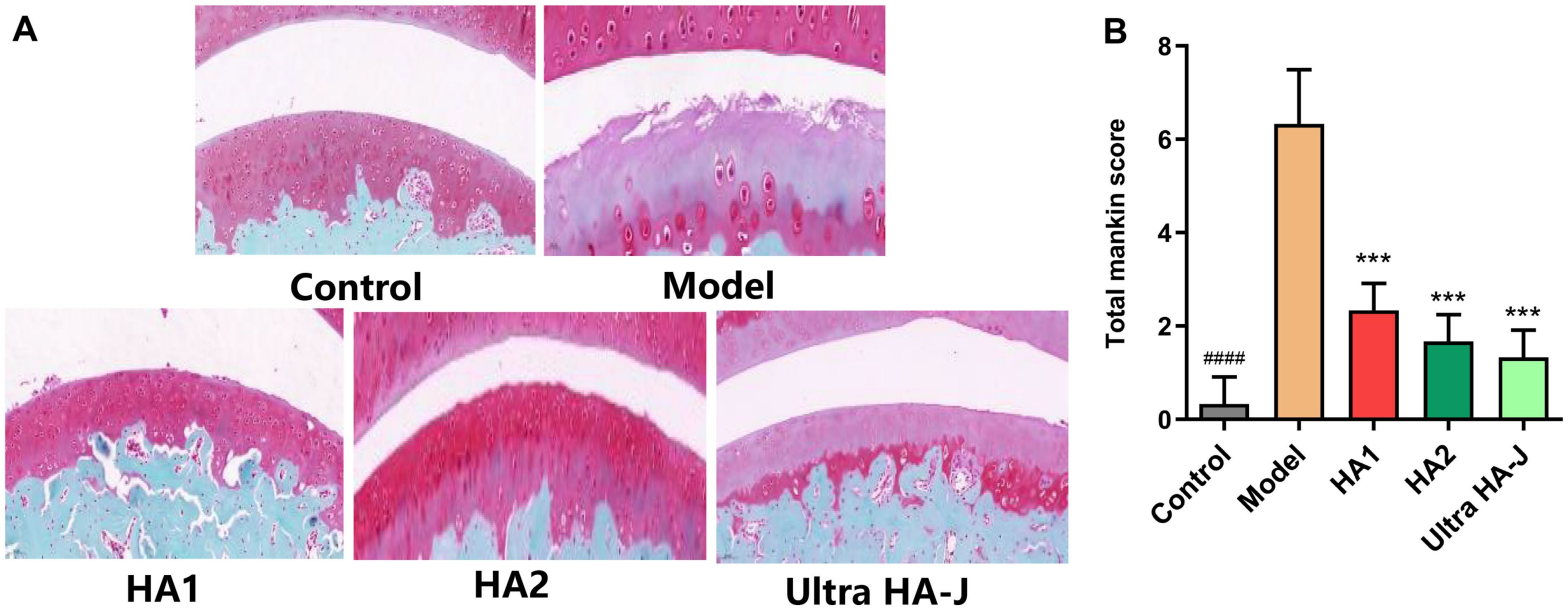
Histological evaluation of joint activity with different groups of H&E staining of MIA-induced OA rats. **(A)** The representative image. **(B)** The combined score for cartilage structure. ####*p* < 0.0001 indicates control group vs. model group; ***p < 0.001 indicates intervention group vs. model group.

As shown in Figure 2A, the joints of intact rats retained intact superficial and smooth articular cartilage surfaces, with an underlying layer of flattened chondrocytes in the tangential zone. In addition, chondrocytes in these joints are normally distributed in the parallel, transitional, and radial zones of the articular cartilage. As expected, rats with OA had irregular surfaces, accompanied by loss of cartilage tissue, degeneration of the articular cartilage, and disappearance of chondrocytes in the tangential, transitional, and radial zones of the cartilage. Notably, supplemental HA altered the histological changes in rats with OA. Elevated Mankin scores in osteoarthritic rats decreased with each HA supplement (Figure 2B), and further decreases were observed with HA2 and Ultra HA-J treatments.

### 3.3 HA regulated inflammatory factors in the serum of MIA-induced OA rats

**Figure 3:**
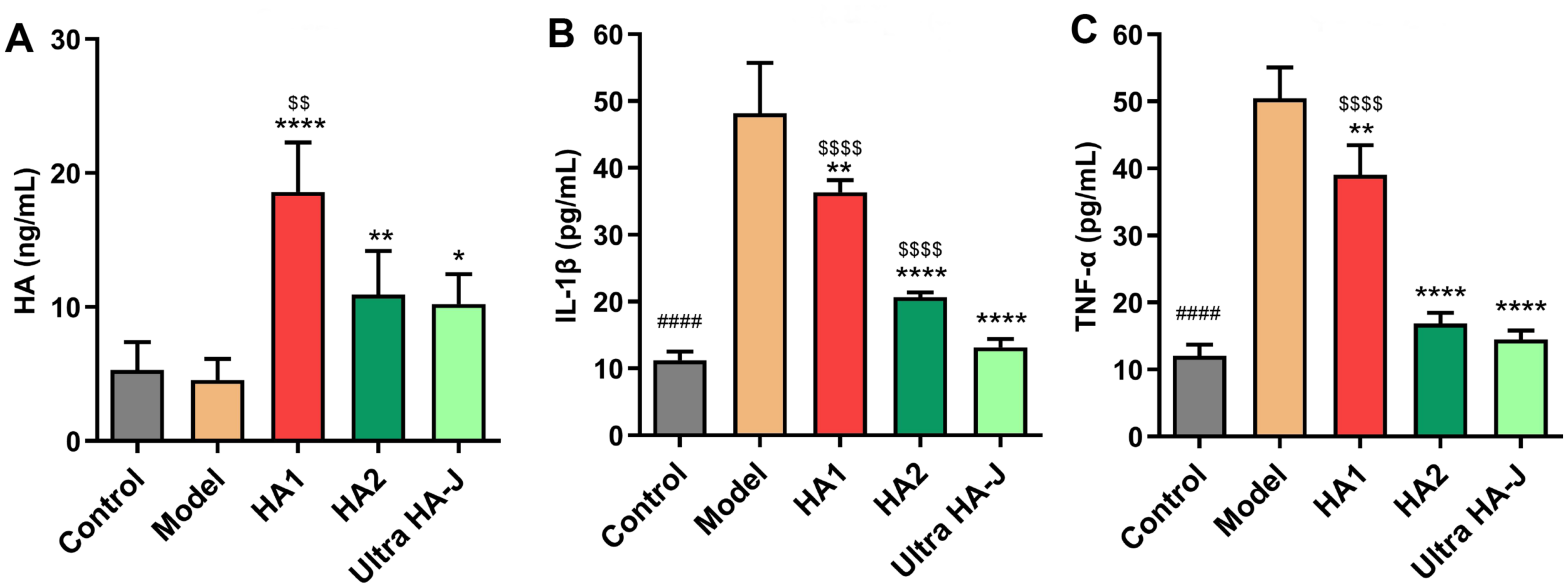
The content of HA **(A)**, IL-1β **(B)**, and TNF-α **(C)** in the serum of MIA-induced OA rats. #### *p* < 0.0001 indicates control group vs. model group; *p < 0.05, **p < 0.01, ****p < 0.0001 indicate intervention group vs. model group; $$*p* < 0.01, $$$$*p* < 0.0001 indicate HA group vs. Ultra HA-J group.

As shown in Figure 3A, the serum HA concentration in the model group was significantly lower than that in the control group. Notably, HA supplementation in rats resulted in a substantial increase in serum HA concentration, with lower-MW HA demonstrating a more pronounced effect in enhancing serum HA levels. Inflammatory response is an important factor associated with OA pathogenesis, and pro-inflammatory cytokines play a prominent role in sustaining tissue injury and chronic inflammation during OA progression. We examined the effect of HA on the production of inflammatory cytokines associated with OA, such as TNF-α and IL-1β, in MIA-induced OA rats. As shown in Figures 3B,C, serum IL-1β and TNF-α concentrations increased in rats with OA compared with control rats. HA supplementation reduced serum concentrations of inflammatory cytokines. As the MW of HA increased, the effect of reducing the concentration of inflammatory cytokines became more significant.

### 3.4 HA regulated inflammatory factors in the synovial fluid of MIA-induced OA rats

**Figure 4:**
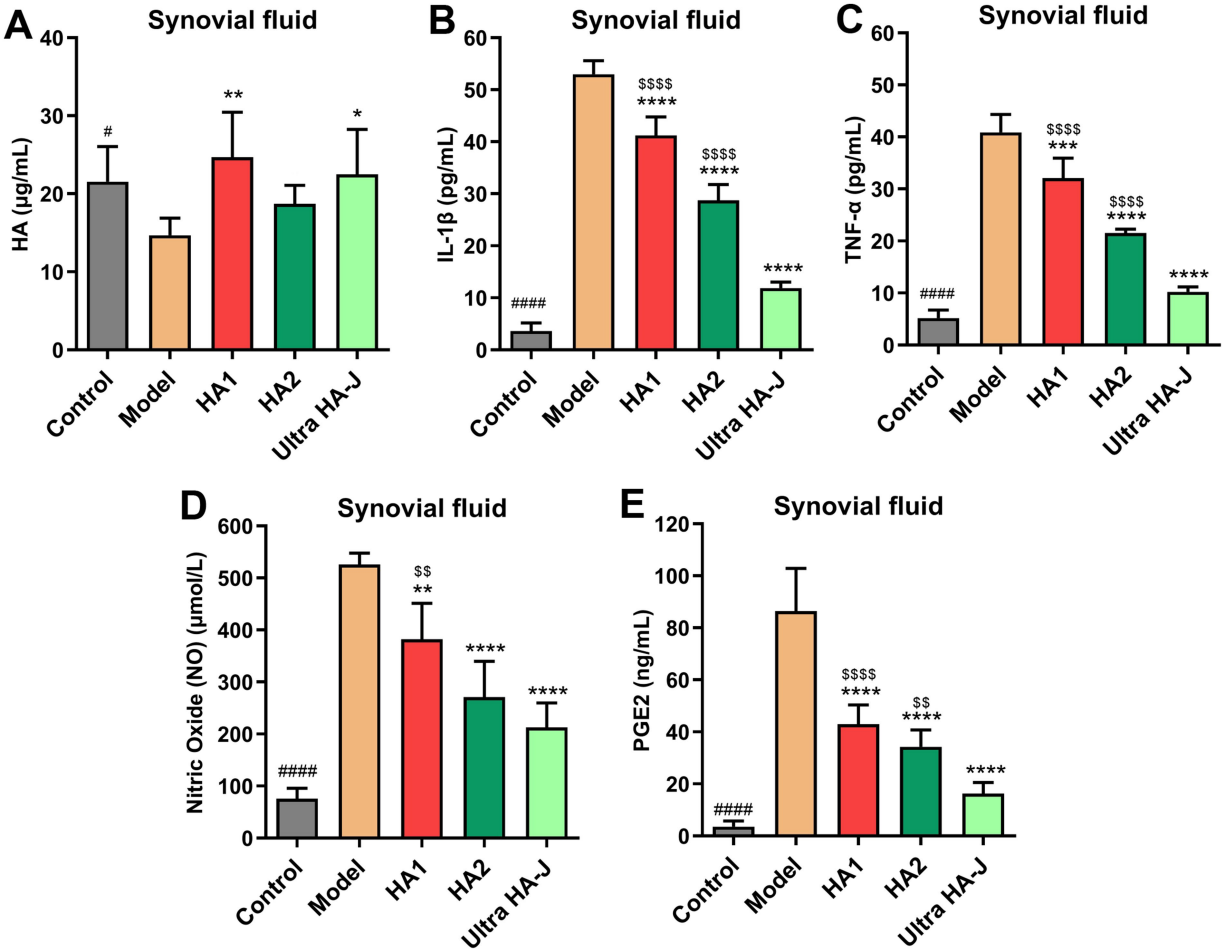
The content of HA **(A)**, IL-1β **(B)**, TNF-α **(C)**, NO **(D)**, and PGE2 **(E)** in the synovial fluid of MIA-induced OA rats. #*p* < 0.05, ####*p* < 0.0001 indicate control group vs. model group; *p < 0.05, **p < 0.01, ****p < 0.0001 indicate intervention group vs. model group; $$*p* < 0.01, $$$$*p* < 0.0001 indicates HA group vs. Ultra-HA-J group.

As shown in Figure 4A, the HA concentration in the synovial fluid was lower in rats with OA than in control rats. Compared with the Model group, supplementation with different forms of HA significantly increased the HA concentration in the synovial fluid. Additionally, the concentrations of IL-1β, TNF-α, NO, and PGE2 in synovial fluid increased in rats with OA compared with control rats (Figures 4B–E). HA supplementation reduced synovial fluid concentrations of inflammatory cytokines. Numerically, Ultra HA-J treatment provided the lowest IL-1β, TNF-α, NO, and PGE2 concentrations compared to other groups with OA, which showed the best anti-inflammatory effect.

### 3.5 HA regulated immune response in the synovial fluid of MIA-induced OA rats

**Figure 5:**
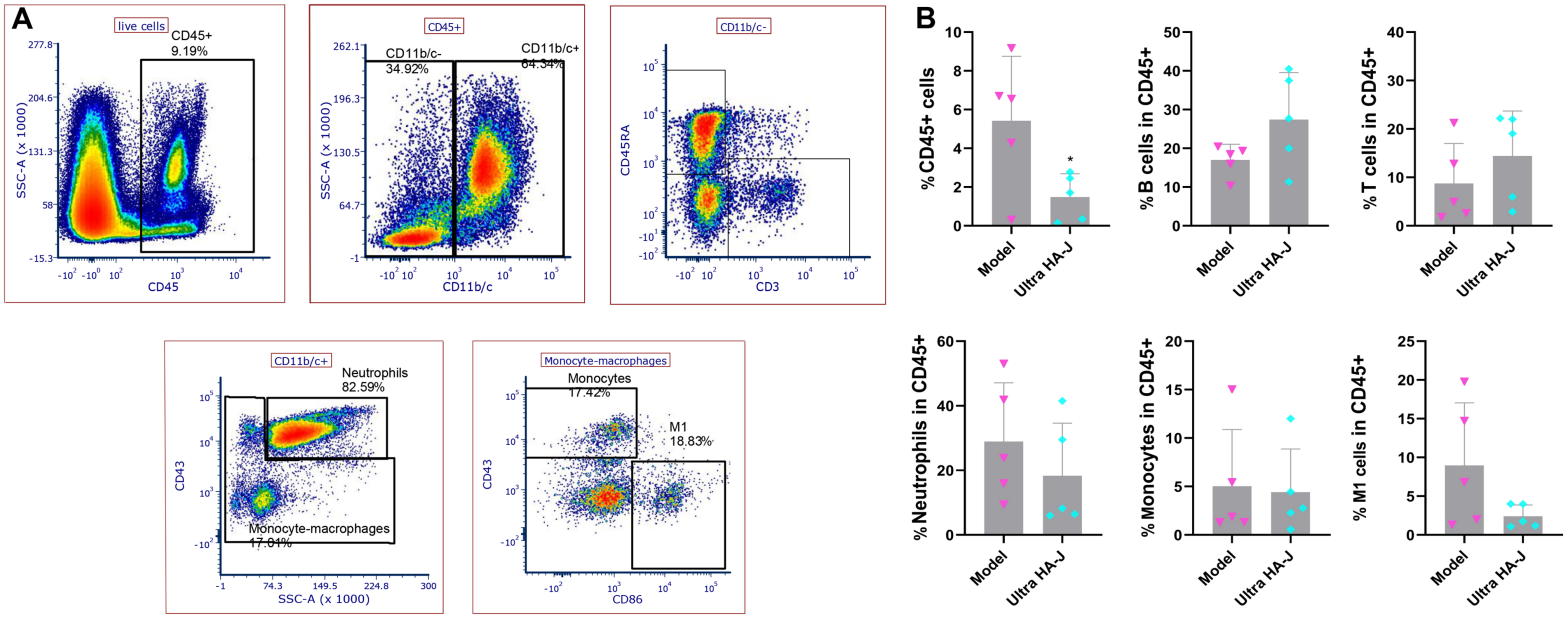
HA changes the frequency of CD45+ subpopulation cells in synovial fluid. **(A)** Gating strategies for CD45++ cell subpopulations in the synovial fluid. **(B)** The frequencies of CD45+ cells, B cells, T cells, Neutrophils, Monocytes, and M1 cells were analyzed using flow cytometry.

Flow cytometry analysis of conventional CD45+ cell subsets in the synovial fluid (Figure 5 and Supplementary Material) showed no significant change in monocyte count between the model and Ultra-HA-J groups. Compared to the model group, the frequency of CD45+ cell subsets, neutrophils, and M1 cells decreased in the Ultra-HA-J group. In addition, we compared the frequencies of B and T cells, which increased in the Ultra-HA-J group.

### 3.6 HA regulated mRNA expression of inflammatory mediators in the articular cartilage tissues of MIA-induced OA rats

**Figure 6:**
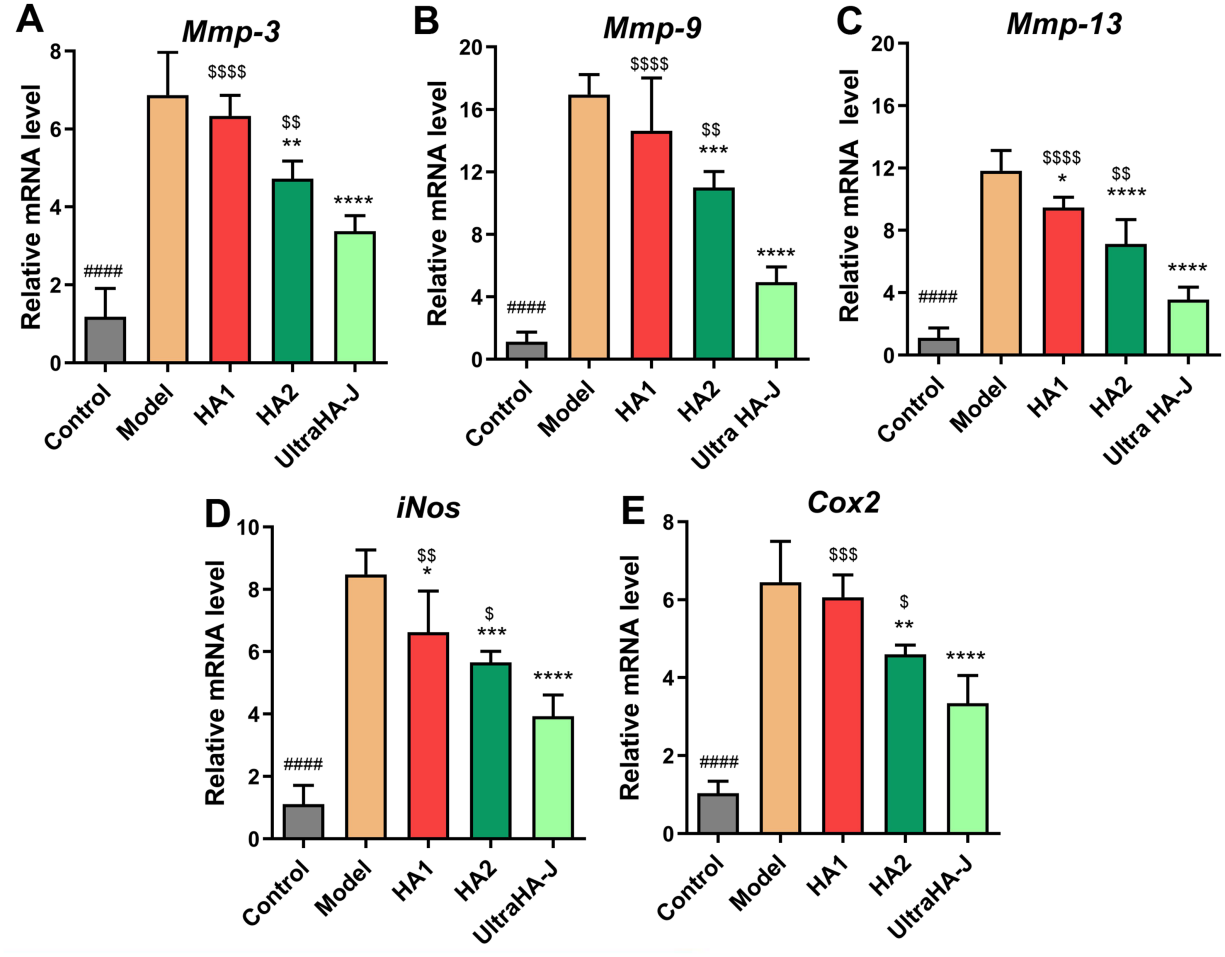
The mRNA expression of **(A)** Mmp-3, **(B)** Mmp-9, **(C)** Mmp-13, **(D)** iNos, and **(E)** Cox2 in the articular cartilage tissues of rats. ####*p* < 0.0001 indicates control group vs. model group; *p < 0.05, **p < 0.01, ***p < 0.001, ****p < 0.0001 indicate intervention group vs. model group; $*p* < 0.05, $$*p* < 0.01, $$$*p* < 0.001, $$$$*p* < 0.0001 indicate HA group vs. Ultra HA-J group.

As shown in Figure 6, the mRNA expression of nitric oxide synthase (iNOS), cyclooxygenase-2 (COX-2), matrix-degrading enzymes, matrix metalloproteinase (MMP)-3, MMP-9, and MMP-13 was higher in the model group than in the control group. In the intervention group, expression of these genes was significantly suppressed. These results suggest that HA inhibits inflammatory responses and articular cartilage damage in rats with MIA-induced OA by inhibiting inflammatory cytokines and MMPs. These inhibitory effects appear to be positively correlated with the molecular weight of HA.

### 3.7 Ultra HA-J significantly improved joint discomfort in clinical trial

**Figure 7:**
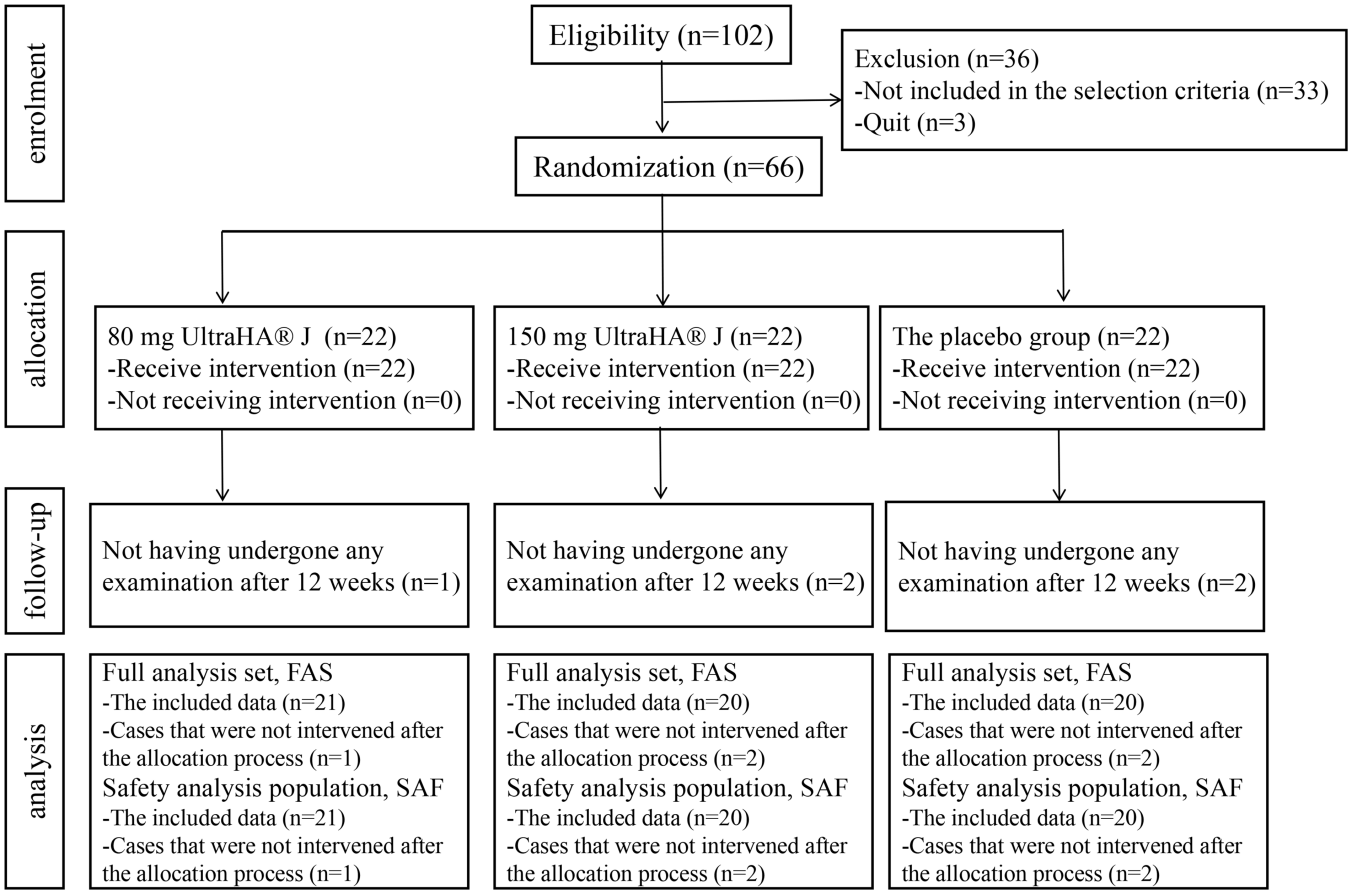
Flow chart of the study design.

**Figure 8:**
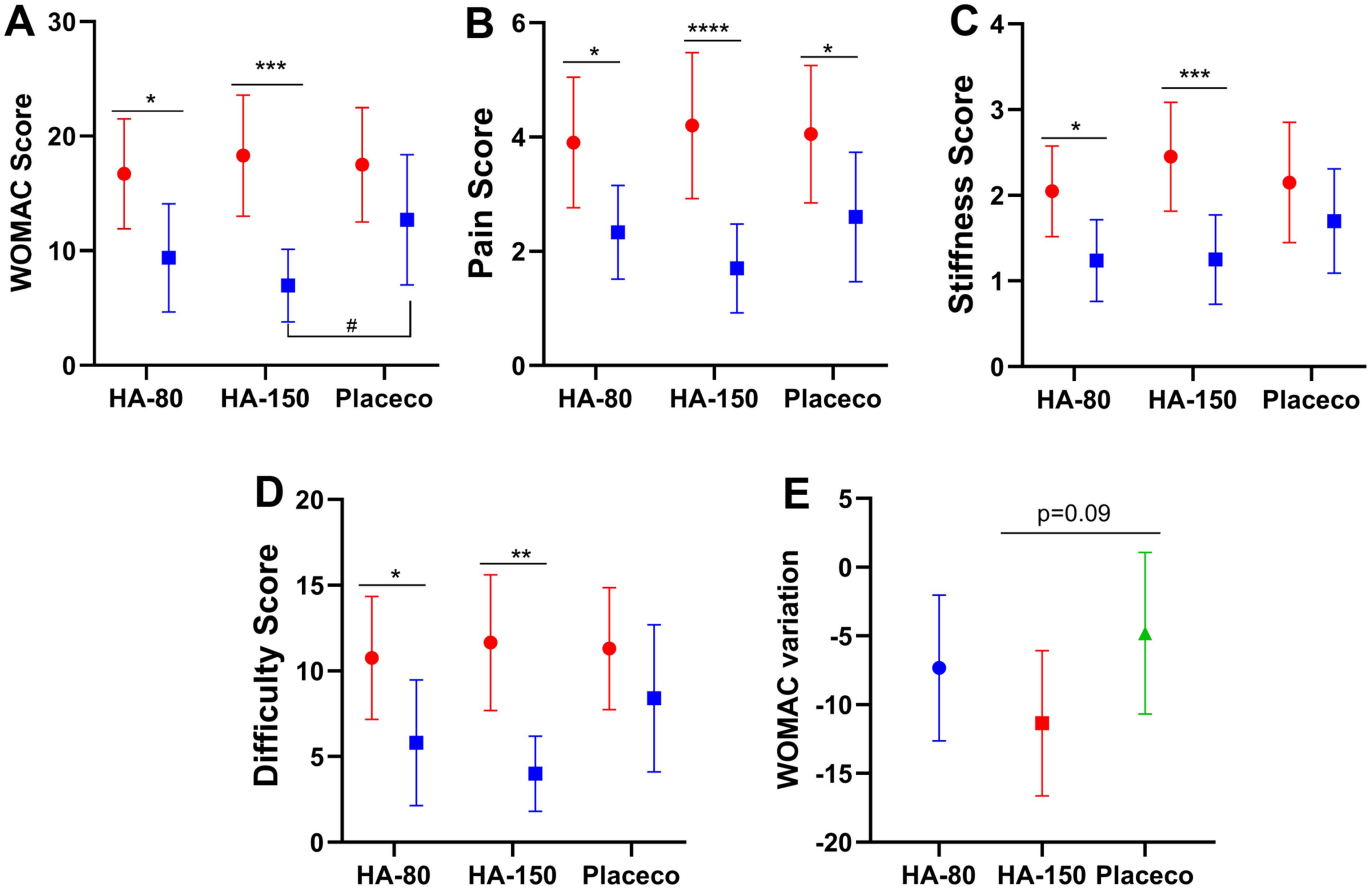
Changes in **(A)** WOMAC score, **(B)** Pain score, **(C)** Stiffness score, **(D)** Difficulty score, and **(E)** WOMAC variation of Clinical Trial. Data are shown as means with 95% confidence intervals (CI) Red line: pre-intervention group; blue line: post-intervention group; *p < 0.05, **p < 0.01, ***p < 0.001, ****p < 0.0001 indicate: pre-intervention group vs. post-intervention group; #*p* < 0.05, HA-150 group vs. placebo group.

66 participants who met the criteria were included in this trial and were randomly assigned to the HA-80 group, HA-150 group, and placebo group, with 22 participants in each group. All participants received the assigned intervention, but it was confirmed that 5 participants did not come for examination after 12 weeks of intake (Figure 7). Since these cases had not received any intervention after being assigned, they were excluded from the analysis. All enrolled subjects completed the trials without any clinically detectable adverse events in either group. Treatment compliance was >90% in both groups. Assessment at each time point was performed according to the WOMAC scoring. As shown in Figure 8, compared to the placebo group, the pain, stiffness, difficulty, and functional WOMAC scores decreased significantly after treatment. Besides, stratified analyses based on background variables (age, sex, and other relevant factors) were performed to examine the relationship between HA and bone function. There are no differences between-group differences in ∆WOMAC, ∆Pain, ∆Stiffness, and ∆Difficulty scores (Supplementary Material).

### 3.8 Safety evaluation

Blood and urine indicators of all participants were tested. The results showed that HA-80 and HA-150 interventions had no significant impact on blood and urine indices compared to the placebo group (Supplementary Material).

## 4 Discussion

OA is the most common form of arthritis. It is characterized by varying degrees of pain and functional impairment, which seriously affects the quality of life of patients and imposes a substantial economic burden. Owing to a paucity of effective strategies for alleviating OA, ongoing research has focused on developing novel, safe, and effective strategies. MIA-induced rodent models of OA are known to show decreased subchondral bone density, sparse trabecular bone, and other osteoporotic manifestations accompanied by inflammation, which are exacerbated over time (23). This method has become widely adopted for constructing animal models of OA. In this study, we established an OA model by injecting MIA into rat knee joints. The results showed that MIA causes significant irregular surfaces accompanied by a loss of cartilage tissue, degeneration of the articular cartilage, and disappearance of chondrocytes in the tangential, transitional, and radial zones of the cartilage. It also causes inflammation of the synovial fluid and immune disorders in rats. These results indicated the successful construction of an OA model. Notably, the intake of HA with different MW can significantly improve all joint injuries induced by MIA, including the repair of pathological tissues and inhibition of the inflammatory response.

Inflammation or injury can increase the permeability of the synovial membrane, leading to an increase in the leakage of plasma fluid and thereby reducing the concentration of HA in the joint. This decrease in HA concentration and/or molecular weight can cause changes in the viscoelasticity of the synovial fluid, resulting in joint dysfunction (24). Under the action of enzymes during free radicals and inflammation, high-molecular-weight hyaluronic acid is decomposed and inhibits the immune response, preventing excessive inflammation. Low-molecular-weight fragments transmit signals related to tissue damage and mobilize immune cells (25). Low-molecular-weight hyaluronic acid (<300 kD) stimulates cell proliferation and initiates pathways involving inflammation, stimulating ciliary beat frequency through RHAMM (HA-mediated motility receptor) (26). In the present study, IL-1β and TNF-α in serum and synovial fluid were significantly increased in OA rats when compared with the control group, indicating inflammation due to OA. Studies have shown that an overproduction of IL-1β is involved in the pathogenesis of OA by mediating chondrocyte inflammation and cartilage degeneration (27–29). IL-1β and TNF-α can stimulate chondrocytes and synovial cells to produce iNOS, which in turn produces NO and promotes chondrocyte apoptosis (30). The content of iNOS, an enzyme responsible for NO production, is increased in the synovial fluid of patients with OA (31), which mediates the production of inflammatory mediators, angiogenesis, and cartilage destruction (32, 33). Excess NO production by iNOS enhances MMP activity and downregulates agglutinin and collagen biosynthesis, leading to cartilage damage (34). PGE2, a major COX-2 product, is highly secreted by osteoblast lineages in the subchondral bone tissue of patients with OA (35). Tissue-specific knockout of COX-2 in the bone cells of OA mice eliminates the production of PGE2 in the subchondral bone, thereby slowing the progression of the disease (36). Coherent results for PGE2 concentration in the synovial fluid were also observed in the present study in rats with OA. The results of inflammation are consistent with those of previous studies, suggesting that these inflammatory factors are involved in physiological changes in OA and are important intervention targets for alleviating OA. Supplementation with HA significantly inhibited the inflammatory response, suggesting that HA may target regulatory validation indicators to alleviate OA.

OA development is accompanied by dramatic changes in the immune system. MMP is a zinc-dependent, potent endopeptidase belonging to a large family of proteases called the Nova superfamily. The levels of MMP-1, MMP-3, and MMP-9 in the synovial fluid were significantly associated with OA severity (37). In human patients with OA undergoing end-stage total knee replacement, high concentrations of macrophages, T cells, and neutrophils are found in the synovial fluid (38). The CD45+ subpopulation is a key immune cell subpopulation critical for the regulation of T-cell receptor signaling. CD45+ cell subsets can differentiate into different T-cell subtypes and perform various functions following antigenic stimulation (39, 40). CD45+ T cells, such as those found in patients with rheumatoid arthritis, are closely related to the body’s systemic REDOX balance, thus affecting immune dysfunction and the occurrence and persistence of inflammatory arthritis (41). This suggests that the CD45+ cell subpopulation is involved in various immune processes in arthritis. CD45 cells can be divided into CD45+ and CD45-, and CD45+ cells can be further divided into CD11b/c- (B- and T-cell subsets) and CD11b/c+. CD11b/c+ cells can be further subdivided into neutrophil and monocyte subsets (monocytes and M1 cells, respectively). Compared to the model group, the frequency of CD45+ cell subsets, neutrophils, and M1 cells decreased in the Ultra-HA-J group. It is worth noting that neutrophils are one of the first cells to reach the site of inflammation and can release a variety of inflammatory mediators, such as reactive oxygen species, to promote inflammation. M1 macrophages also secrete pro-inflammatory cytokines, such as TNF-α and IL-1β, which aggravate inflammation. In addition, we compared the frequencies of B and T cells, which increased in the Ultra-HA-J group. Together, these findings suggest that arthritis disrupts synovial immunity in rats and that Ultra-HA-J can restore immune impairment by alleviating inflammation and promoting adaptive immunity.

Pain is an important clinical feature of OA and a major complaint in patients with OA (42). In this study, the WOMAC score, pain score, stiffness score, difficulty score, and WOMAC variation were important indicators for measuring the severity of joint discomfort. In this study, animal experiments were employed to evaluate the effects of different molecular weights of HA on OA and to identify the molecular weight that was most effective in alleviating OA. Based on this, a clinical trial was conducted to further verify the clinical efficacy of the selected HA. The results demonstrated that although all molecular weights of HA showed certain OA alleviation effects, Ultra HA-J exhibited the best results, including Joint Swelling, Joint Mankin score, and inflammatory indicators. Based on the above experimental results, Ultra HA-J was ultimately used for the evaluation of clinical efficacy. Although our research results cannot clearly demonstrate the impact of molecular weight on human efficacy, they still can provide some reference basis for the potential applications of this molecular weight of HA in food supplements and functional foods.

Importantly, in this study, the result showed that HA supplementation in rats resulted in a substantial increase in serum HA concentration, with lower-MW HA demonstrating a more pronounced effect in enhancing serum HA levels, but with the least therapeutic benefit, which suggested that the high-MW HA may exert its effects through other mechanisms. A previous study has shown that symbiotic bacteria in the human body, such as *Bacteroides*, *Bifidobacterium*, *Dialister*, and *Faecalibacterium* can metabolize HA, thereby generating large amounts of SCFAs, including acetic acid, propionic acid, and butyric acid (43). The gut microbiota itself can alleviate inflammatory arthritis (44), and oral administration of SCFAs has also been demonstrated to ameliorate experimental rheumatoid arthritis in part by the function of increasing IL-10-producing B cells (45). Notably, the MW of HA is also crucial for its interactions with toll-like receptor 2 (TLR2) and TLR4 (46), whereas TLR is a putative pathway linking maternal inflammation and neurodevelopmental disorders (47). Moreover, several data indicate that HA, as a glycosaminoglycan commonly present in the extracellular matrix, may be involved in controlling the functions of epithelial cells, immune cells, and nerve cells by interacting with different types of cells in the intestinal microenvironment (46). These findings suggested that the efficacy of HA may also result from local immunomodulation in the gut, HA metabolites, or size-dependent interactions with tissues and receptors.

In summary, oral HA may be a potential nutritional strategy for improving OA injuries and has shown excellent clinical results. Importantly, this experiment still has some shortcomings: (1) There is a lack of sufficient data to clarify the absorption, distribution, and interaction mechanism between oral HA and regional bone and joint tissues in the human body. Therefore, in future research, we will consider using molecular markers and other techniques to deeply explore the metabolic process of HA in the human body; (2) Further pharmacokinetic studies (such as Cmax, t½, conjugation patterns) were conducted on rats and humans to examine the effects and safety of different doses of HA, to elucidate the dose-dependent effects and safety of HA.

## Data Availability

The original contributions presented in the study are included in the article/Supplementary Material, further inquiries can be directed to the corresponding authors.
