# Supplementary material for: Role of oral hyaluronic acid for joint health: insights from rat models and clinical trials
Source: Front Nutr. 2025 Dec 17;12:1691328. doi: 10.3389/fnut.2025.1691328 (PMC12754907; doi:10.3389/fnut.2025.1691328)
Supplement: Supplementary file 1 [file Table_1.pdf]

Item	HA-80								HA-150								Placeco							
	n	Mean	SD	Med	Min	Max	95% CI-	95% CI+	n	Mean	SD	Med	Min	Max	95% CI-	95% CI+	n	Mean	SD	Med	Min	Max	95% CI-	95% CI+
White Blood Cell Count (WBC) /µl	21	5110	1330	5000	3000	8000	4504	5715	20	5125	1522	4800	3100	9600	4413	5837	20	5540	1414	5300	3000	9100	4878	6202
Red Blood Cell Count (RBC) ×10^	21	453	36	449	398	519	437	470	20	452	44	460	369	552	431	473	20	458.3	40	454	379	561	439	477
Hemoglobin (Hb) g/dL	21	13.8	1.1	13.6	12.2	16.2	13.3	14.3	20	13.7	0.9	13.7	11.1	15.2	13.3	14.1	20	13.9	1.3	13.9	11.7	16.7	13.3	14.5
Hematocrit (Ht) %	21	43.4	3.4	43.2	37.2	50	41.9	45	20	43.4	2.7	43.4	36.4	48.1	42.1	44.7	20	43.4	3.6	44	36.8	49.4	41.7	45
Platelet Count (PLT) ×10^4/µL	21	25.4	6.4	25.8	13.9	40.1	22.5	28.3	20	24.6	5.3	23.6	16.7	36.9	22.1	27.1	20	25.3	5	25.2	16.6	35.5	22.9	27.6
Aspartate Aminotransferase (AST) U	21	20	4	20	13	30	18	22	20	21	10	19	10	58	16	25	20	20.5	5	22	12	33	18	23
Alanine Aminotransferase (ALT) U	21	17	5	17	8	28	15	20	20	17	11	14	5	55	11	22	20	20.4	12	17	8	54	15	26
Gamma-Glutamyl Transpeptidase (	21	27	23	22	8	119	16	38	20	21	15	16	10	77	14	29	20	27.7	32	19	8	160	12	43
Total Bilirubin (T-BIL) mg/dL	21	0.9	0.5	0.8	0.4	2.2	0.7	1.1	20	0.8	0.3	0.8	0.4	1.5	0.7	0.9	20	0.8	0.2	0.8	0.5	1.3	0.7	0.9
Total Protein (TP) g/dL	21	7.1	0.3	7.1	6.4	7.6	6.9	7.2	20	7.1	0.3	7	6.6	8	7	7.2	20	7	0.3	7	6.5	7.6	6.9	7.2
Urea Nitrogen (UN) mg/dL	21	14.6	4.1	13.4	9.6	26.3	12.8	16.5	20	13.2	3.1	12.4	9.6	21	11.8	14.7	20	12.5	3.2	13	6.8	18.7	11	14.1
Creatinine (CRE) mg/dL	21	0.77	0.21	0.76	0.45	1.12	0.67	0.86	20	0.75	0.13	0.73	0.58	1.12	0.68	0.81	20	0.7	0.18	0.67	0.45	1.17	0.65	0.82
Uric Acid (UA) mg/dL	21	5.2	1.3	5.2	3.4	8.4	4.6	5.8	20	5.2	1.2	5.5	2.7	7	4.7	5.8	20	5.1	1.1	5.3	3.2	6.8	4.6	5.6
Sodium (Na) mEq/L	21	142	1	142	139	143	141	142	20	142	2	141	138	145	141	143	20	140.5	2	140	137	143	140	141
Potassium (K) mEq/L	21	4.1	0.3	4.1	3.5	4.6	4	4.2	20	4.2	0.3	4.2	3.7	4.7	4	4.3	20	4	0.3	4.1	3.4	4.5	3.9	4.2
Chloride (Cl) mEq/L	21	104	2	104	101	106	103	104	20	103	1	104	100	106	103	104	20	102.6	2	102	99	106	102	104
Serum Amylase (AMY/S) U/L	21	68	20	72	33	105	59	77	20	75	36	73	34	215	58	92	20	74.3	23	68	52	157	63	85
Total Cholesterol (T-Cho) mg/dL	21	207	28	210	156	266	194	220	20	200	36	202	145	269	183	216	20	206.4	29	203	132	246	193	220
HDL-Cholesterol (HDL-Cho) mg/d	21	63	16	62	35	104	56	70	20	63	13	61	31	87	57	69	20	64.2	15	64	34	89	57	71
LDL-Cholesterol (LDL-Cho) mg/dL	21	121	26	124	77	169	110	133	20	116	36	116	59	192	99	133	20	119.7	27	119	61	169	107	132
Triglycerides (TG) mg/dL	21	101	59	87	29	247	74	128	20	92	69	73	26	347	59	124	20	102.7	62	68	41	244	74	132
Glucose (GLU) mg/dL	21	93	16	87	71	139	86	100	20	93	14	89	78	142	86	99	20	94.7	22	89	75	180	84	105
Hemoglobin A1c (HbA1c: NGSP)	21	5.6	0.7	5.4	5.1	7.6	5.3	6	20	5.5	0.2	5.5	5.1	5.9	5.4	5.6	20	5.4	0.3	5.4	4.8	5.9	5.3	5.5

n:Number of cases; Mean:Mean value; SD:Standard deviation; Med:Central value; Min:Minimum value; Max:Maximum value; 95%CI-:Lower limit of 95% confidence interval; 95%CI+:Upper limit of 95% confidence interval

Urine tests

Item	Judgement	HA-80		HA-150		Placeco	
		n	Percentage of cases (%)	n	Percentage of cases (%)	n	Percentage of cases (%)
Urine Protein	(-)	21	100	19	95	20	100
	(±)	0	0	1	5	0	0
	(+)	0	0	0	0	0	0
	(2+)	0	0	0	0	0	0
	(3+)	0	0	0	0	0	0
Urine Glucose	(-)	19	90.5	20	100	20	100
	(±)	0	0	0	0	0	0
	(+)	1	4.8	0	0	0	0
	(2+)	0	0	0	0	0	0
	(3+)	1	4.8	0	0	0	0
Urine pH	< 5.0	0	0	0	0	0	0
	5.0 ~ 7.5	21	100	20	100	20	100
	> 7.5	0	0	0	0	0	0
Urine Occult Blood	(-)	19	90.5	20	100	20	100
	(±)	1	4.8	0	0	0	0
	(+)	1	4.8	0	0	0	0
	(2+)	0	0	0	0	0	0
	(3+)	0	0	0	0	0	0

Individual score list for each item of WOMAC (Yellow: Not Measured / NA: Not Available)

Group	Sex	Age	ID		WOMAC score	Pain score	Stiffness score	Difficulty score		WOMAC score	Pain score	Stiffness score	Difficulty score
HA-80	male	54	85649	VISIT1	43	10	4	29	VISIT2	12	4	1	7
	male	38	85653	VISIT1	20	6	4	10	VISIT2	3	1	2	0
	female	49	85663	VISIT1	5	2	2	1	VISIT2	2	0	1	1
	male	59	85665	VISIT1	13	3	2	8	VISIT2	11	3	1	7
	female	80	85667	VISIT1	27	6	1	20	VISIT2	27	4	3	20
	female	48	85728	VISIT1	16	7	2	7	VISIT2	3	1	1	1
	male	62	85750	VISIT1	20	4	2	14	VISIT2	8	2	2	4
	male	73	85751	VISIT1	9	2	2	5	VISIT2	41	7	3	31
	male	59	85933	VISIT1	31	8	3	20	VISIT2	26	6	3	17
	female	43	85936	VISIT1	12	3	2	7	VISIT2	4	3	1	0
	female	55	85939	VISIT1	3	1	0	2	VISIT2	2	1	0	1
	male	39	85940	VISIT1	11	3	2	6	VISIT2	4	2	0	2
	male	26	85947	VISIT1	11	4	2	5	VISIT2	6	3	1	2
	female	52	85951	VISIT1	5	1	1	3	VISIT2	4	2	1	1
	female	66	85962	VISIT1	11	2	1	8	VISIT2	0	0	0	0
	female	67	85976	VISIT1	36	5	4	27	VISIT2	14	2	2	10
	male	72	85979	VISIT1	24	4	3	17	VISIT2	11	1	2	8
	male	45	86149	VISIT1	9	1	1	7	VISIT2	2	1	0	1
	female	57	86185	VISIT1	21	6	0	15	VISIT2	11	3	0	8
	male	43	86458	VISIT1	13	1	3	9	VISIT2	4	1	2	1
	female	61	86499	VISIT1	11	3	2	6	VISIT2	2	2	0	0
HA-150	male	51	85604	VISIT1	7	3	2	2	VISIT2	3	2	1	0
	male	62	85650	VISIT1	16	3	3	10	VISIT2	4	1	1	2
	female	54	85654	VISIT1	11	1	4	6	VISIT2	1	0	1	0
	male	71	85659	VISIT1	16	2	3	11	VISIT2	5	1	1	3
	female	53	85882	VISIT1	25	7	0	18	VISIT2	10	3	0	7
	male	54	85884	VISIT1	47	7	5	35	VISIT2	0	0	0	0
	male	46	85885	VISIT1	9	2	1	6	VISIT2	9	1	2	6
	female	44	85934	VISIT1	28	6	4	18	VISIT2	21	4	4	13
	female	52	85949	VISIT1	15	3	1	11	VISIT2	1	0	0	1
	male	59	85950	VISIT1	7	2	2	3	VISIT2	10	3	2	5
	male	62	85954	VISIT1	11	1	1	9	VISIT2	0	0	0	0
	female	37	85956	VISIT1	6	2	2	2	VISIT2	5	3	1	1
	female	55	85958	VISIT1	9	3	3	3	VISIT2	2	0	2	0
	female	58	86186	VISIT1	11	4	1	6	VISIT2	6	2	1	3
	male	41	86251	VISIT1	27	9	4	14	VISIT2	1	1	0	0
	male	67	86410	VISIT1	24	5	3	16	VISIT2	21	3	2	16
	female	55	86456	VISIT1	9	2	1	6	VISIT2	2	0	0	2
	female	49	86459	VISIT1	31	10	2	19	VISIT2	19	6	2	11
	male	57	86465	VISIT1	37	8	4	25	VISIT2	10	3	2	5
	female	49	86467	VISIT1	20	4	3	13	VISIT2	9	1	3	5
Placeco	male	47	85602	VISIT1	52	11	5	36	VISIT2	31	7	4	20
	male	45	85603	VISIT1	11	4	1	6	VISIT2	4	0	1	3
	female	44	85644	VISIT1	29	10	4	15	VISIT2	22	6	2	14
	female	63	85645	VISIT1	12	2	2	8	VISIT2	9	2	1	6
	female	55	85655	VISIT1	26	5	4	17	VISIT2	26	6	4	16
	female	43	85656	VISIT1	8	2	0	6	VISIT2	46	8	2	36
	female	43	85658	VISIT1	16	5	1	10	VISIT2	1	0	0	1
	female	44	85672	VISIT1	22	4	2	16	VISIT2	1	1	0	0
	female	60	85723	VISIT1	3	1	0	2	VISIT2	4	1	1	2
	male	50	85938	VISIT1	11	4	3	4	VISIT2	2	2	0	0
	male	60	85941	VISIT1	25	3	4	18	VISIT2	16	1	3	12
	male	76	85945	VISIT1	7	4	0	3	VISIT2	2	0	1	1
	male	53	85948	VISIT1	24	4	3	17	VISIT2	10	3	2	5
	male	49	85959	VISIT1	14	4	0	10	VISIT2	12	3	2	7
	male	57	85961	VISIT1	18	6	3	9	VISIT2	19	3	4	12
	male	74	86020	VISIT1	15	2	3	10	VISIT2	2	1	1	0
	female	60	86252	VISIT1	10	3	2	5	VISIT2	8	2	2	4
	female	58	86462	VISIT1	17	3	2	12	VISIT2	3	1	0	2
	male	62	86468	VISIT1	19	3	2	14	VISIT2	25	4	2	19
	female	70	86496	VISIT1	11	1	2	8	VISIT2	11	1	2	8

Background of the Test Participants							
		ITT			FAS, SAF		
Item	-	80 mg UltraHA® J	150 mg UltraHA® J	The placebo group	80 mg UltraHA® J	150 mg UltraHA® J	The placebo group
	n	22	22	22	21	20	20
Gender	Male	11 (50%)	11 (50%)	11 (50%)	11 (52.4%)	10 (50%)	10 (50%)
	Female	11 (50%)	11 (50%)	11 (50%)	10 (47.6%)	10 (50%)	10 (50%)
	n	22	22	22	21	20	20
Age	Mean (SD)	54.4 (13.0)	53.8 (8.1)	55.1 (99)	54.7 (13.2)	53.8 (8.3)	55.7 (10.2)
	Med	54.5	54	54	55	54	56
	Min-Max	26-80	37-71	43-76	26-80	37-71	43-76
	n	22	22	22	21	20	20
Height (cm)	Mean (SD)	163.6 (7.1)	166.8 (8.4)	162.0 (7.4)	163.8 (7.2)	166.3 (8.5)	162.3 (7.6)
	Med	163	168.8	162.45	164.9	166.7	162.65
	Min-Max	153.0-174.5	150.8-181.6	144.4 (175.2)	153.0-174.5	150.8-181.6	144.4-175.2
	n	22	22	22	21	20	20
Weight (kg)	Mean (SD)	68.7 (7.8)	70.2 (8.4)	67.2 (8.2)	68.9 (7.9)	69.5 (7.9)	67.7 (8.4)
	Med	69	70.05	64.9	69.7	70.05	66.45
	Min-Max	55.9-86.0	54.2-87.6	54.6-84.2	55.9-86.0	54.2-82.5	54.6-84.2
	n	22	22	22	21	20	20
BMI (kg/m2)	Mean (SD)	25.6 (1.9)	25.2 (2.0)	25.5 (1.6)	25.6 (1.9)	25.1 (1.9)	25.6 (1.6)
	Med	24.9	24.6	25.45	24.9	24.6	25.9
	Min-Max	23.1-29.9	23.1-29.5	23.0-29.0	23.1-29.9	23.1-29.5	23.0-29.0
Systolic blood	n	22	22	22	21	20	20
pressure	Mean (SD)	127.1 (15.3)	127.0 (15.8)	128.2 (17.6)	126.7 (15.6)	126.6 (15.1)	128.4 (17.4)
(mmHg)	Med	127	123.5	125.5	124	123.5	125.5
	Min-Max	105-163	104-158	102-157	105-163	104-158	102-157
Expanded	n	22	22	22	21	20	20
period blood	Mean (SD)	81.7 (8.6)	83.0 (11.8)	83.1 (12.3)	81.5 (8.7)	82.3 (11.7)	82.7 (12.1)
pressure	Med	84	80	83.5	82	79	83.5
(mmHg)	Min-Max	69-100	62-105	48-102	69-100	62-105	48-102
	n	22	22	22	21	20	20
KL	Normal	13 (59.1%)	14 (63.6%)	14 (63.6%)	13 (61.9%)	12 (60.0%)	13 (65.0%)
	Suspicious minor bone	9 (40.9%)	8 (36.4%)	8 (36.4%)	8 (38.1%)	8 (40.0%)	7 (35.0%)

n: Number of cases/Mean: Average value/SD: Standard deviation/Med: Median/Min: Minimum value/Max: Maximum value/ITT: Intention to treat/FAS: Full analysis set/SAF: Safety analysis

Summary of Scores for WOMAC

Item		80 mg UltraHA® J						150 mg UltraHA® J						The placebo group					
		n	Mean	SD	Med	Min	Max	n	Mean	SD	Med	Min	Max	n	Mean	SD	Med	Min	Max
WOMAC	VISIT1 *	21	16.7	10.6	13	3	43	20	18.3	11.3	15.5	6	47	20	17.5	10.7	15.5	3	52
	VISIT2	21	9.4	10.3	4	0	41	20	7	6.7	5	0	21	20	12.7	12.2	9.5	1	46
Pain score	VISIT1 *	21	3.9	2.5	3	1	10	20	4.2	2.7	3	1	10	20	4.1	2.6	4	1	11
	VISIT2 #	21	2.3	1.8	2	0	7	20	1.7	1.7	1	0	6	20	2.6	2.4	2	0	8
Stiffness score	VISIT1 *	21	2	1.2	2	0	4	20	2.5	1.4	2.5	0	5	20	2.2	1.5	2	0	5
	VISIT2 #	21	1.2	1	1	0	3	20	1.3	1.1	1	0	4	20	1.7	1.3	2	0	4
Difficulty score	VISIT1 *	21	10.8	7.9	8	1	29	20	11.7	8.5	10.5	2	35	20	11.3	7.6	10	2	36
	VISIT2 #	21	5.8	8.1	2	0	31	20	4	4.7	2.5	0	16	20	8.4	9.2	5.5	0	36

n: Number of cases/Mean: Average value/SD: Standard deviation/Med: Median/Min: Minimum value/Max: Maximum value.

Summary Table of VAS

Item		80 mg UltraHA® J						150 mg UltraHA® J						The placebo group					
		n	Mean	SD	Med	Min	Max	n	Mean	SD	Med	Min	Max	n	Mean	SD	Med	Min	Max
pain (mm)	VISIT1 *	21	46.5	23.1	54	2	72	20	46.6	19.5	48.5	15	75	20	40.4	23.4	36	10	79
	VISIT2 #	21	26.8	23.1	17	0	68	20	27	24.2	17.5	0	70	20	29.4	23.9	22.5	2	69
Knee stiffness	VISIT1 *	21	42.1	23.2	47	1	72	20	42.1	23.3	46	3	75	20	36.9	23.8	31.5	2	84
	VISIT2 #	21	28.7	24.7	32	0	67	20	26.2	23.3	18	2	70	20	29.8	25.7	25.5	1	75
Knee discomfo	VISIT1 *	21	55	22.7	62	5	81	20	51.4	26.2	59.5	5	91	20	47	24.1	50	3	88
	VISIT2 #	21	32.5	26.4	23	0	70	20	27.5	25.8	16.5	2	75	20	31	22.1	27	3	71
The health	VISIT1 *	21	55.5	22.4	63	4	80	20	51.1	20.4	54.5	12	86	20	47	24.5	49.5	10	92
	VISIT2 #	21	30.6	25.5	27	0	75	20	27	25.7	16.5	0	75	20	26.5	21.4	22.5	3	71

n: Number of cases / Mean: Average value / SD: Standard deviation / Med: Median / Min: Minimum value / Max: Maximum value.

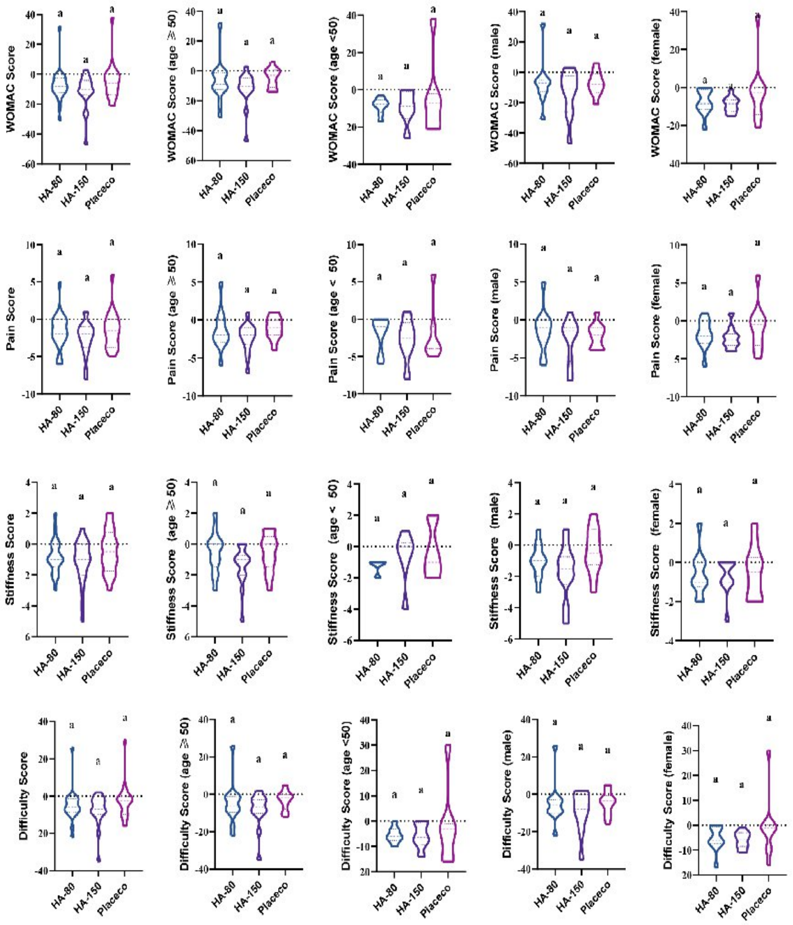

Supplementary Figure 1. Stratified analyses of the roles of HA in bone function based on background variables (age, BMI, baseline WOMAC scores, and other relevant factors) to estimate between-group differences in ΔWOMAC, ΔPain, ΔStiffn

The underlying data, especially for ELISA

	Serum			Synovial fluid				
	IL-1β (pg/	TNF-α (pg	HA (ng/m	IL-1β (pg/	TNF-α (pg	HA (μg/m	NO (μmol	PGE2 (ng/mL)
Control_1	12.763	10.445	4.317	1.722	3.166	19.243	86.780	4.688
Control_2	10.505	14.028	3.951	5.278	6.725	20.436	99.492	2.390
Control_3	12.132	12.619	8.855	5.696	5.470	18.095	63.898	1.266
Control_4	9.508	12.993	4.081	2.804	3.697	29.373	80.848	6.871
Control_5	11.326	10.275	5.804	2.804	6.487	20.576	47.797	2.339
Model_1	33.660	51.964	4.763	52.804	43.932	14.002	491.017	112.473
Model_2	47.801	43.798	4.029	51.069	38.319	13.253	530.848	71.603
Model_3	53.043	57.762	7.410	50.548	37.947	12.288	551.186	81.618
Model_4	51.215	48.631	3.559	53.709	37.872	17.327	543.559	98.056
Model_5	54.797	49.762	2.875	57.804	45.945	16.615	509.661	69.635
HA1_1	34.269	43.464	18.279	44.311	36.529	28.942	355.424	41.501
HA1_2	37.318	34.674	22.447	44.568	34.618	29.627	345.254	36.314
HA1_3	37.496	39.150	15.067	39.826	30.032	27.220	398.644	35.602
HA1_4	36.342	37.851	20.239	41.370	32.391	16.001	316.441	49.915
HA1_5	36.452	40.383	16.477	35.938	26.761	21.553	494.407	51.327
HA2_1	21.295	19.117	9.403	29.096	20.814	18.314	168.983	28.906
HA2_2	19.708	16.041	7.332	32.612	21.374	15.603	287.627	40.980
HA2_3	20.597	16.842	12.159	25.384	22.568	20.825	307.966	38.625
HA2_4	21.110	15.404	14.833	27.890	21.374	20.171	318.136	28.474
HA2_5	20.701	17.328	10.896	28.822	21.499	19.696	271.027	34.639
Ultra HA-	12.459	15.323	14.141	13.795	10.743	15.666	203.729	11.469
Ultra HA-	12.609	12.275	9.897	11.713	10.964	20.710	233.390	17.504
Ultra HA-	14.525	14.542	8.918	10.856	9.944	29.779	157.119	13.340
Ultra HA-	11.817	14.580	9.407	11.915	8.634	27.012	187.627	22.683
Ultra HA-	14.496	15.775	8.834	11.042	10.743	19.183	280.848	16.208
