# Supplementary figures and images for: Role of oral hyaluronic acid for joint health: insights from rat models and clinical trials
Source: Front Nutr. 2025 Dec 17;12:1691328. doi: 10.3389/fnut.2025.1691328 (PMC12754907; doi:10.3389/fnut.2025.1691328)

Model\_1

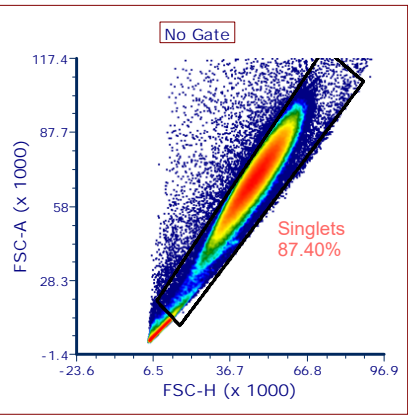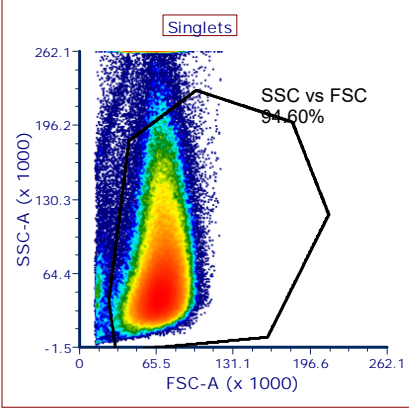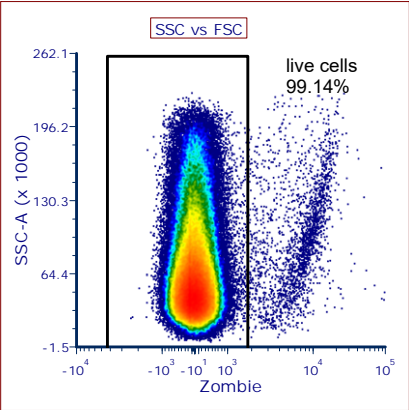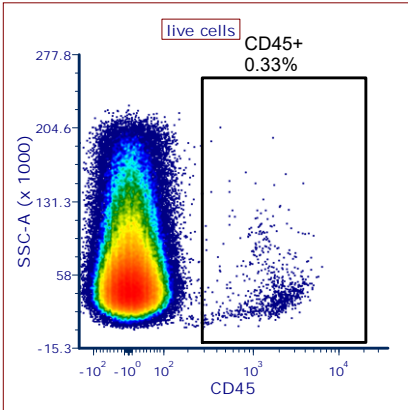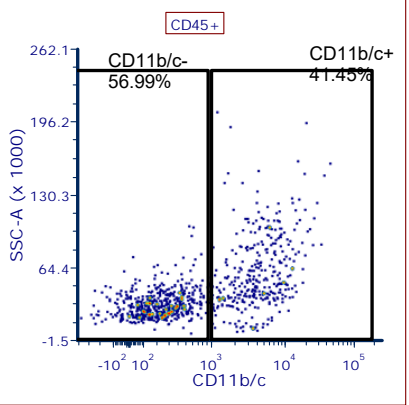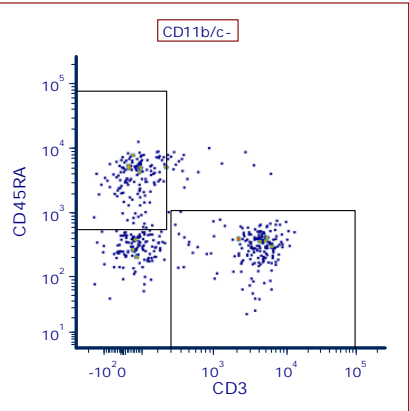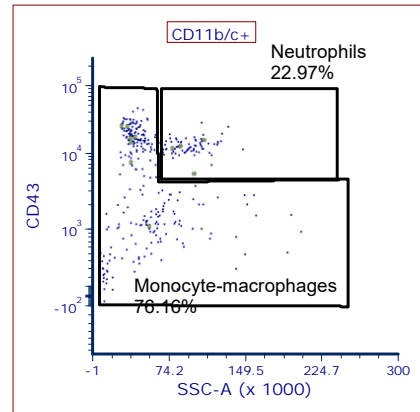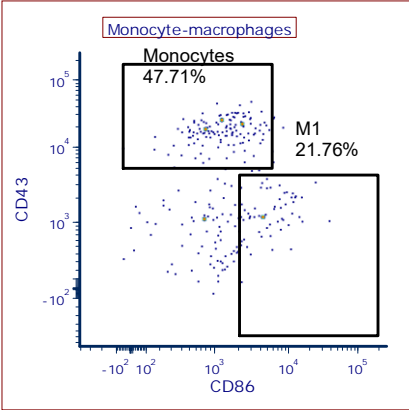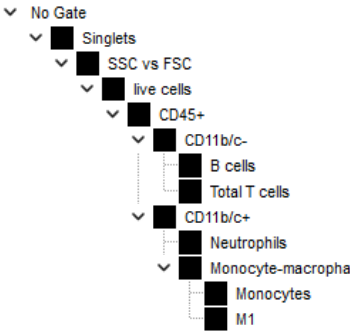

Model\_2

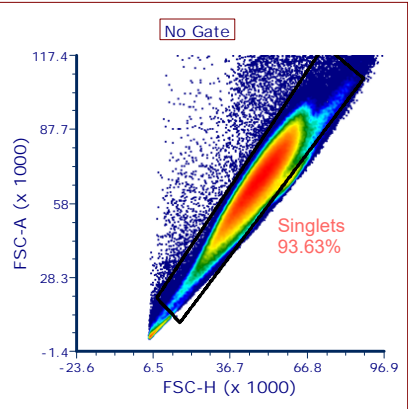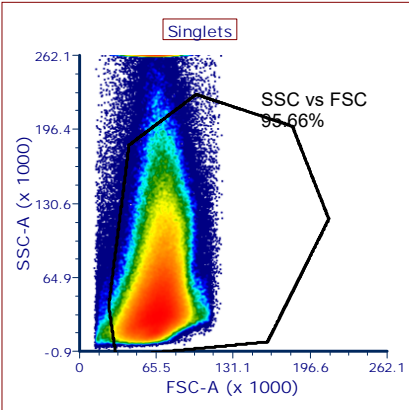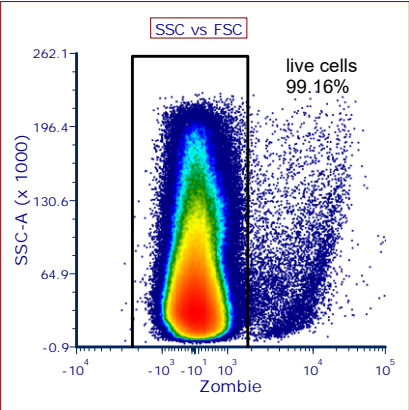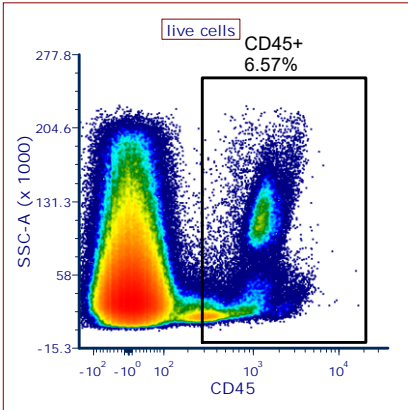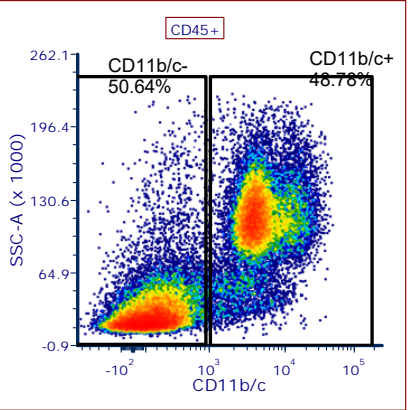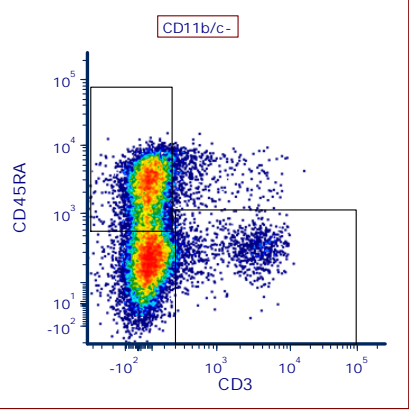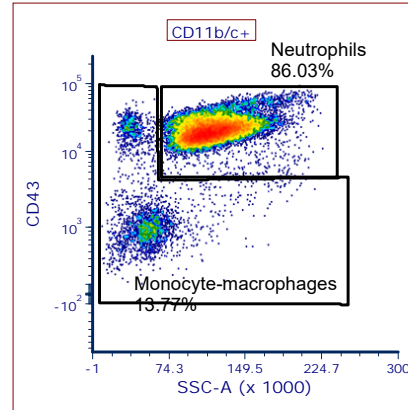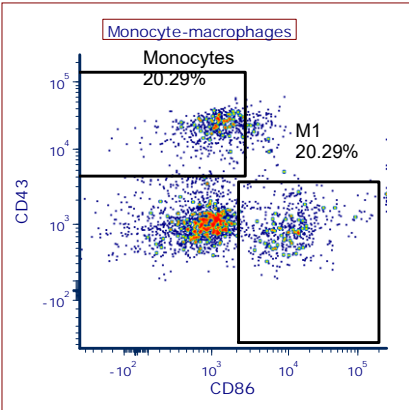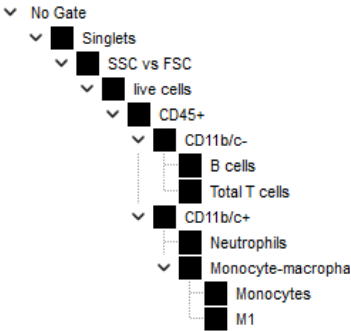

Model\_3

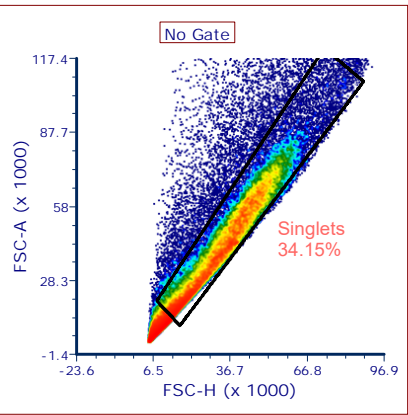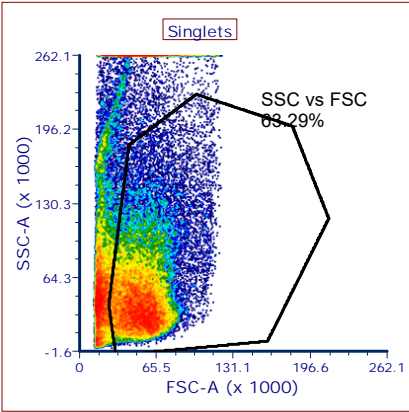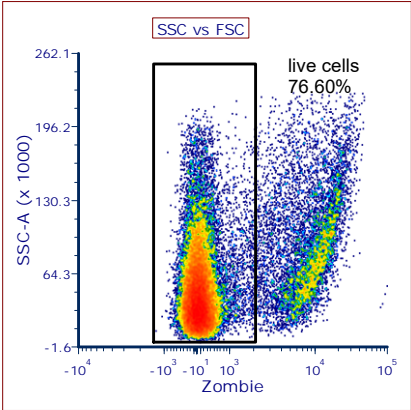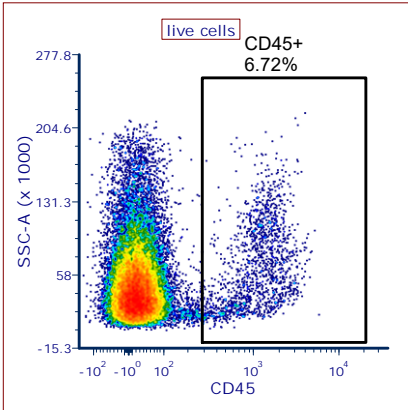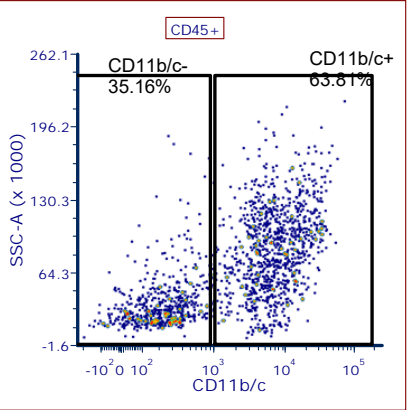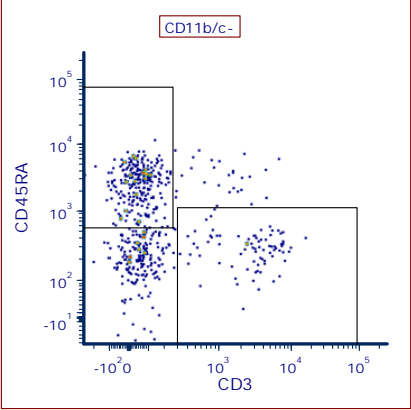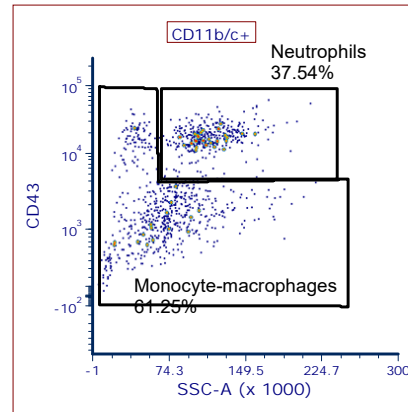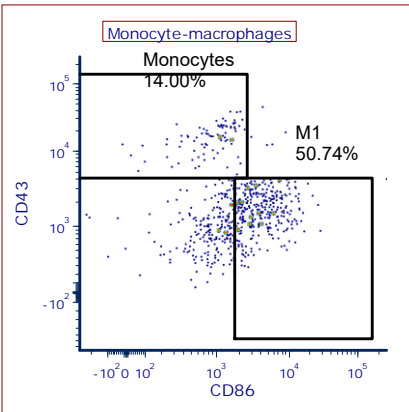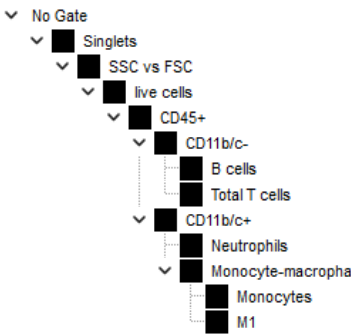

Model\_4

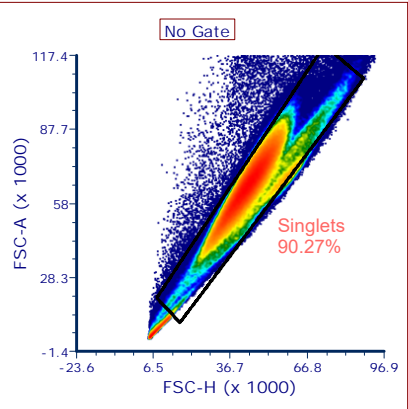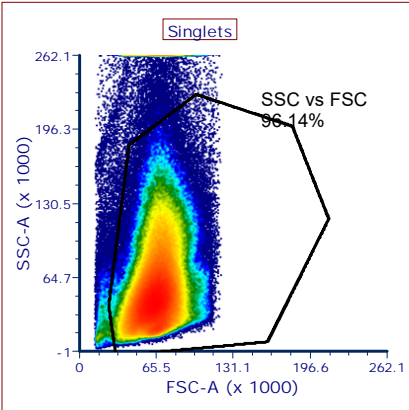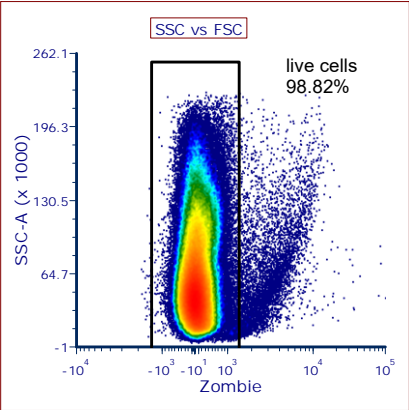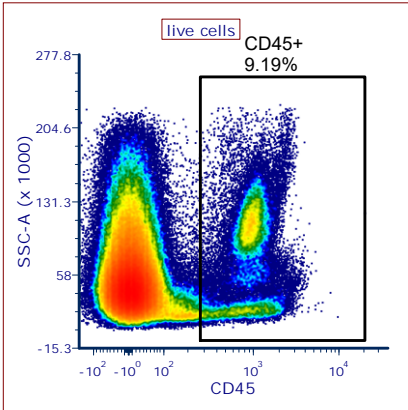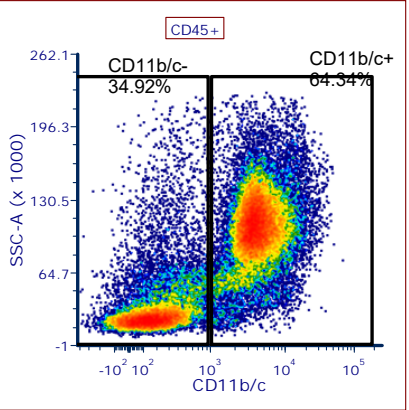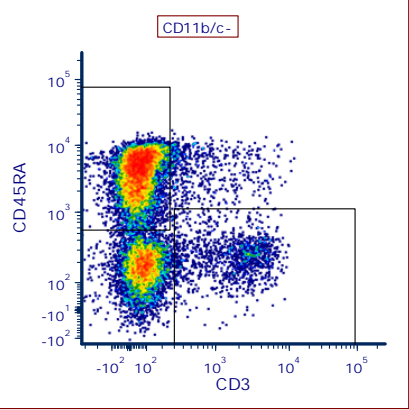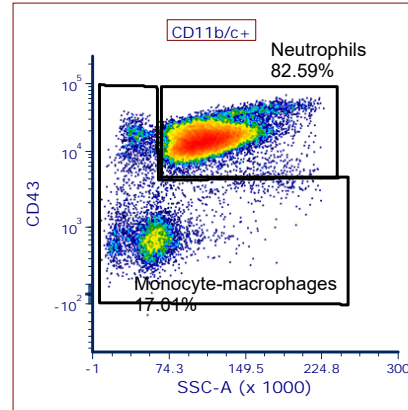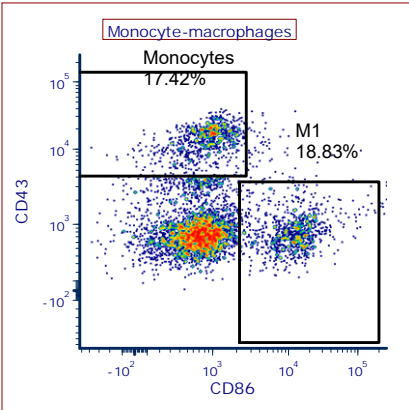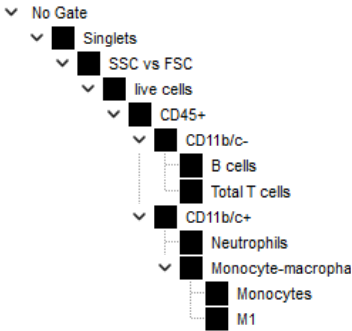

Model\_5

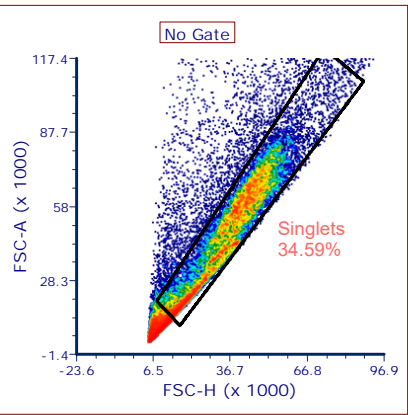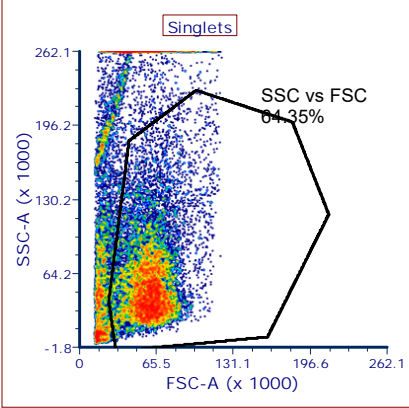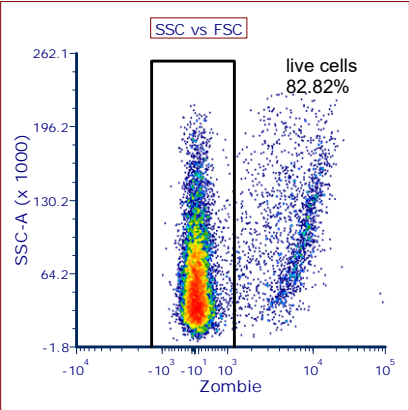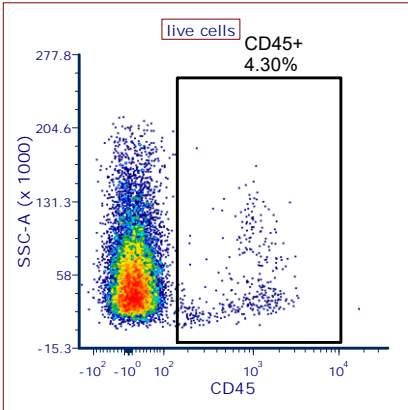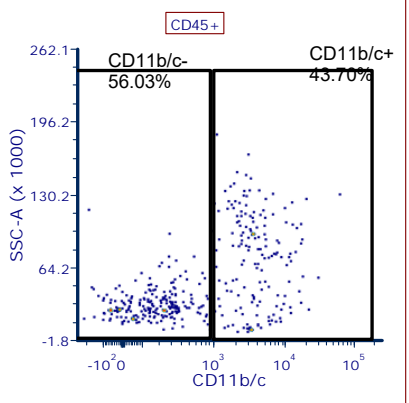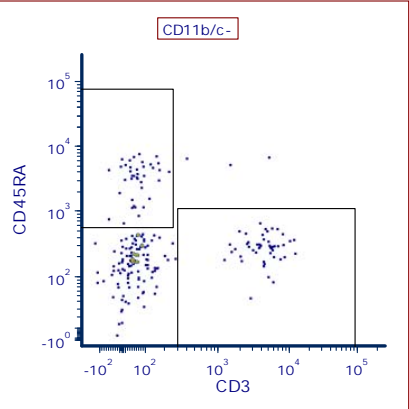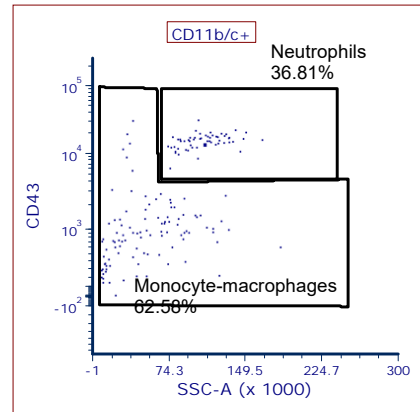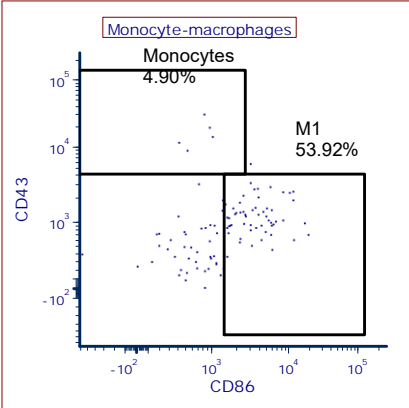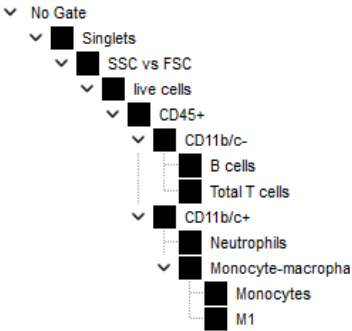

Ultra\_HA-J\_1

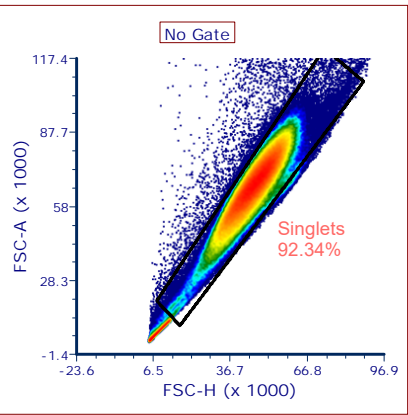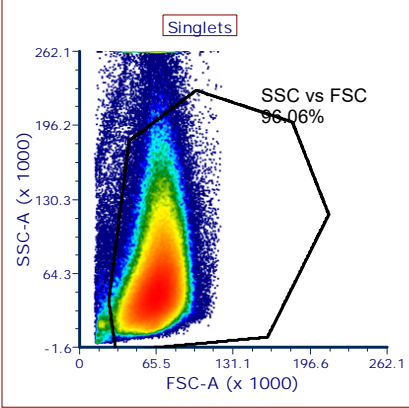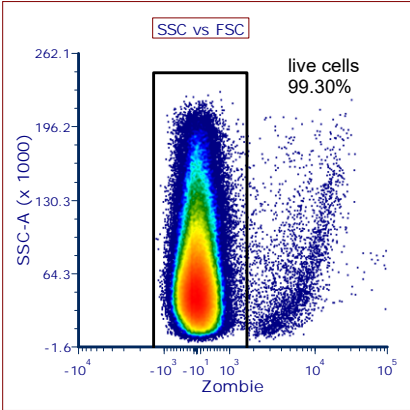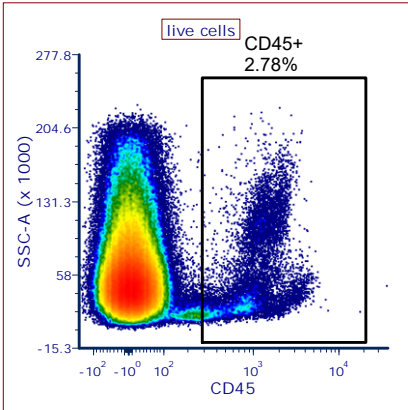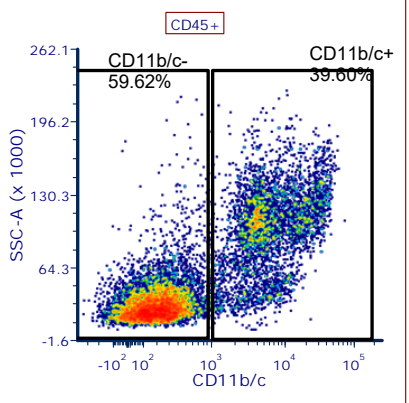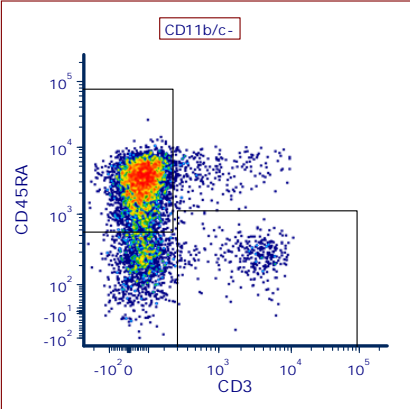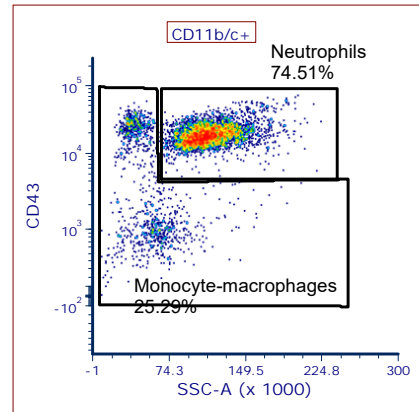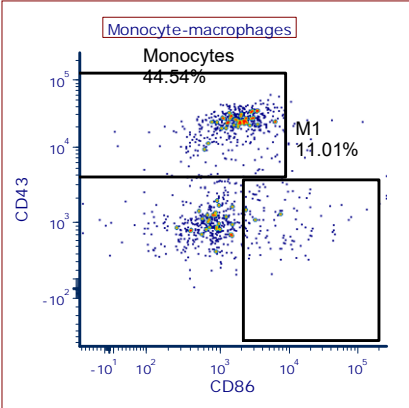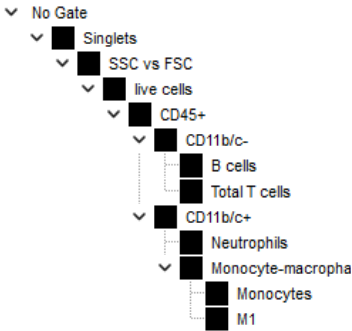

Ultra\_HA-J\_2

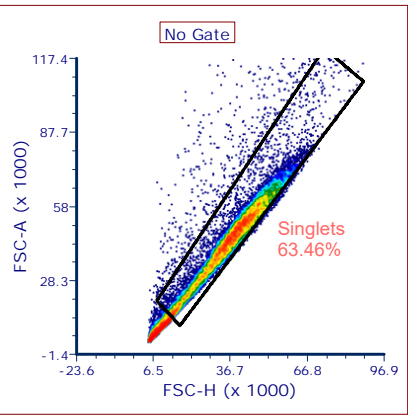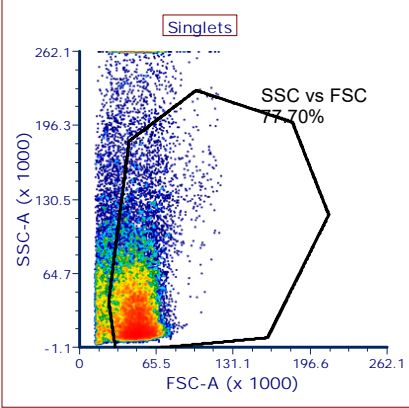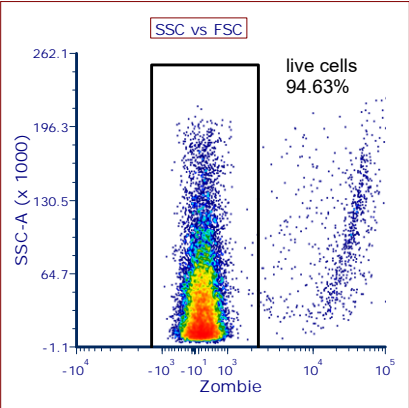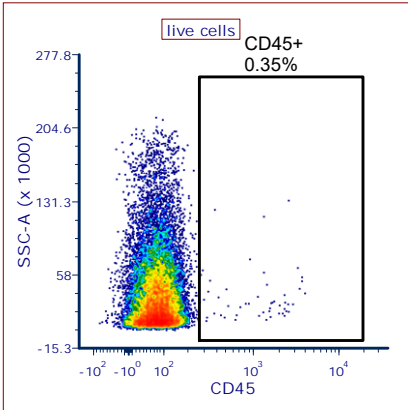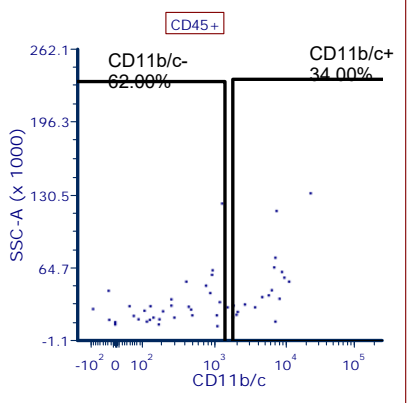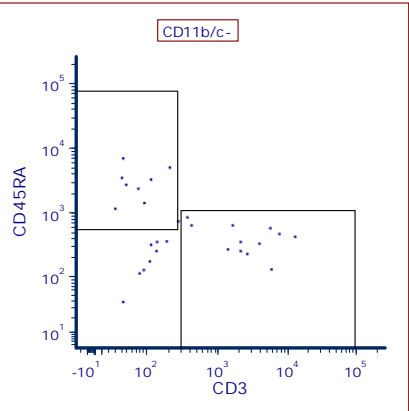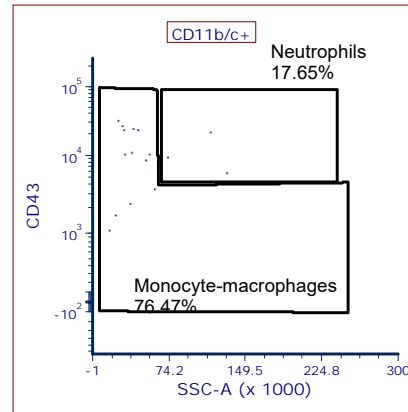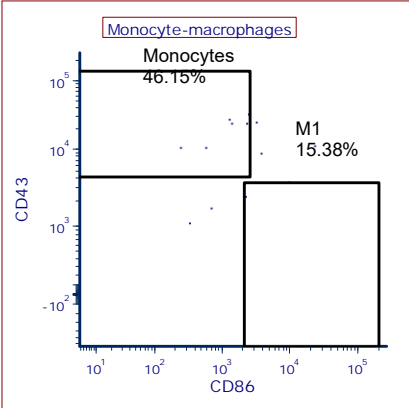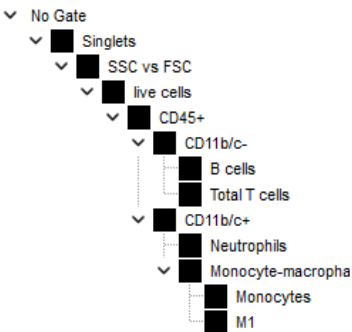

Ultra\_HA-J\_3

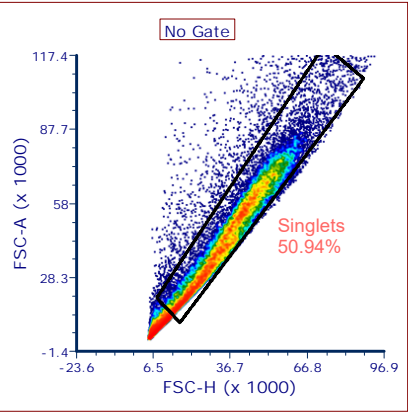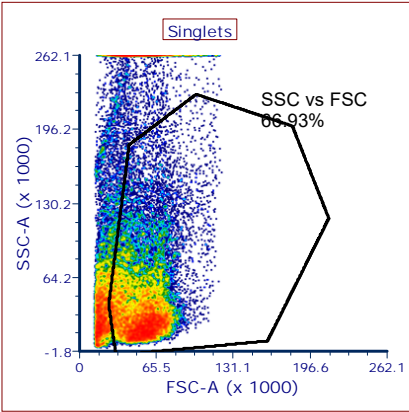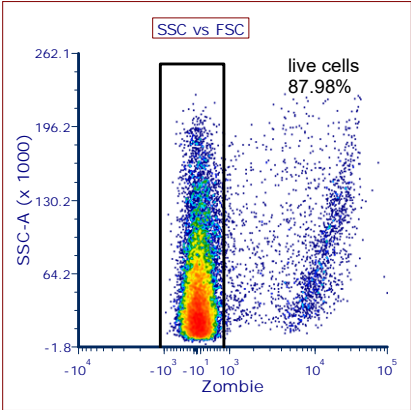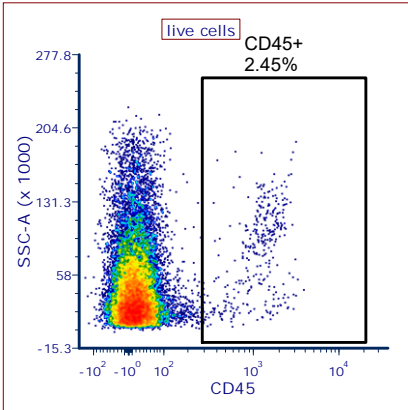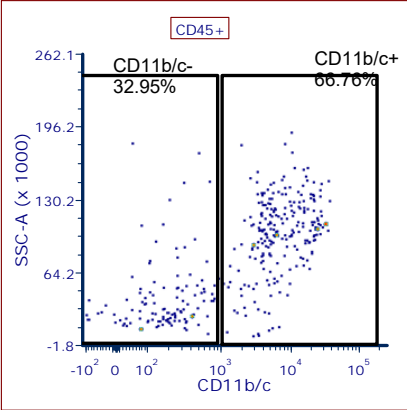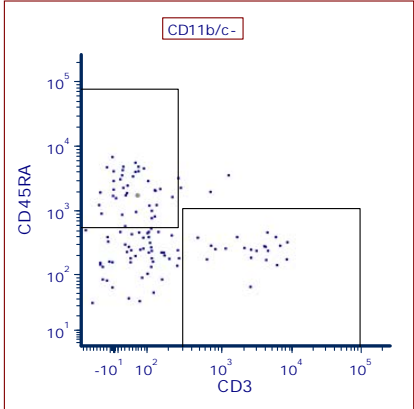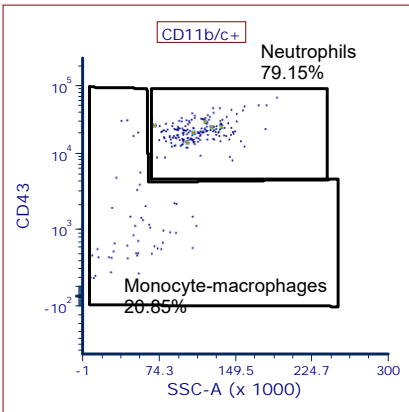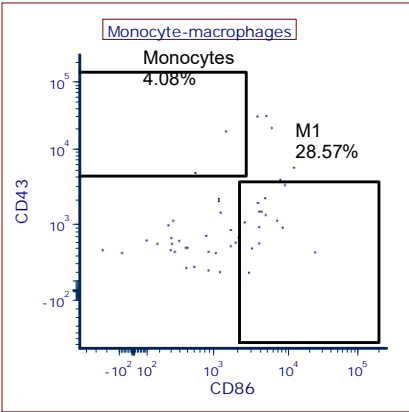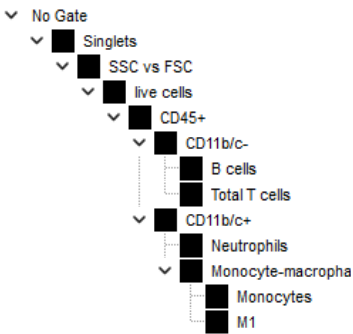

Ultra\_HA-J\_4

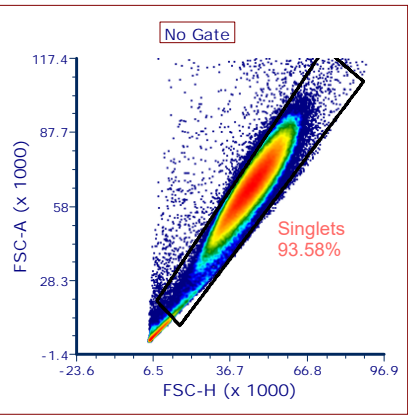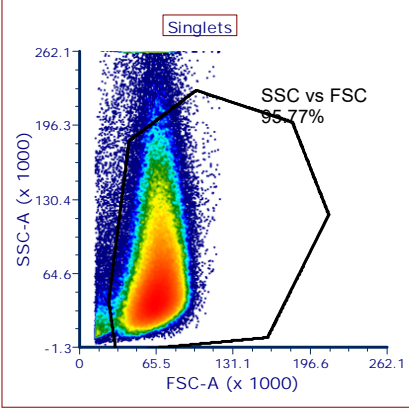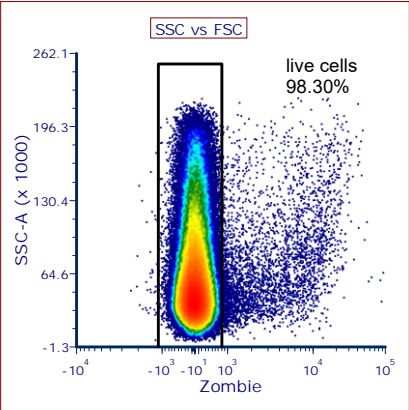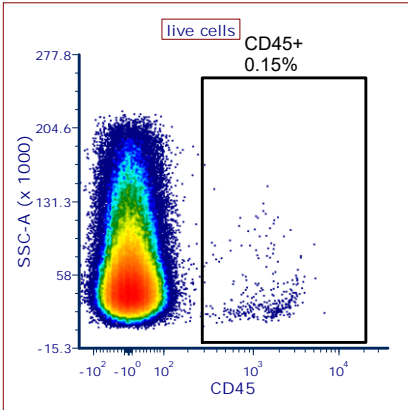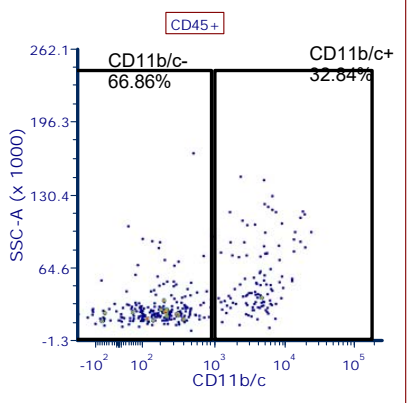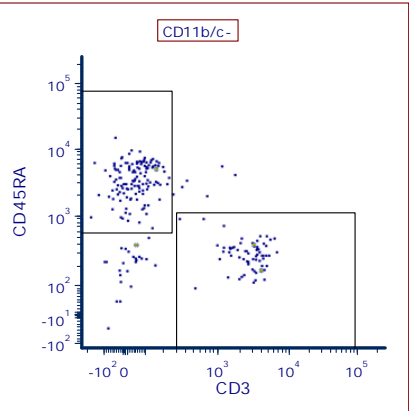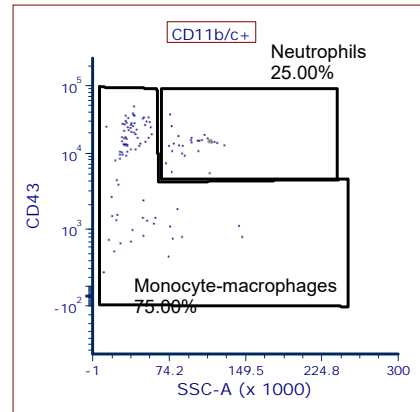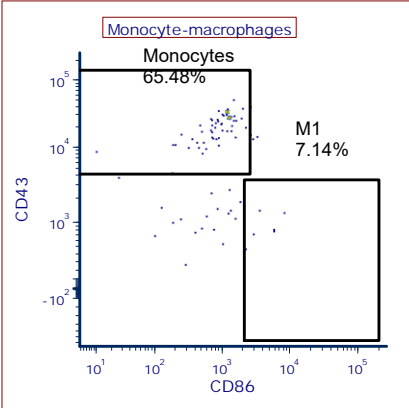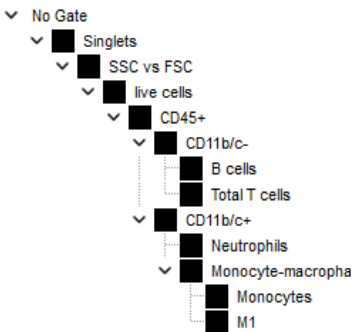

Ultra\_HA-J\_5

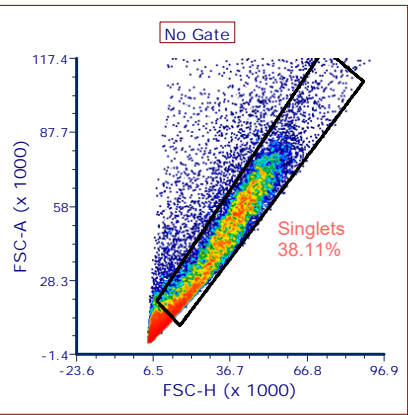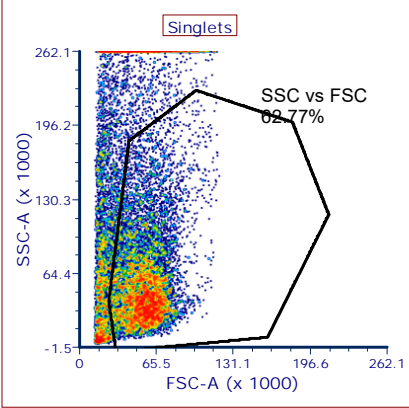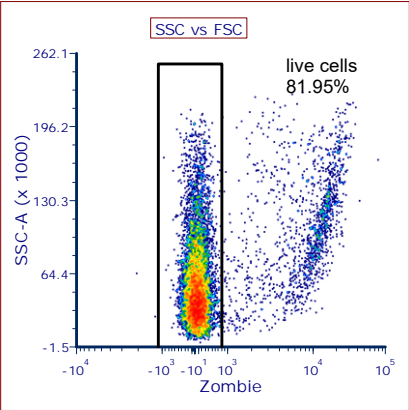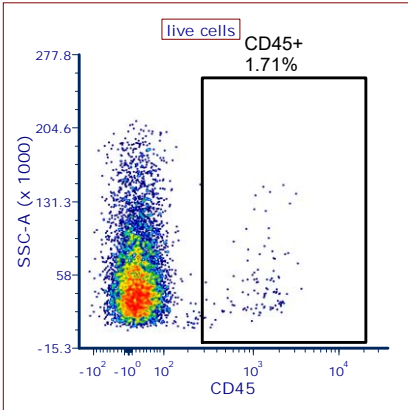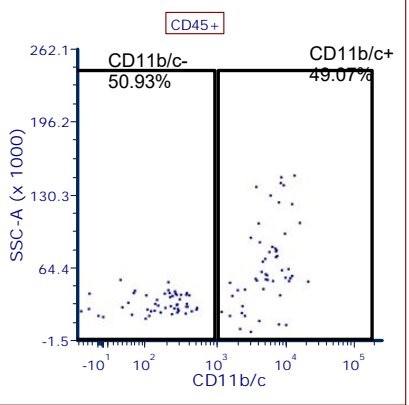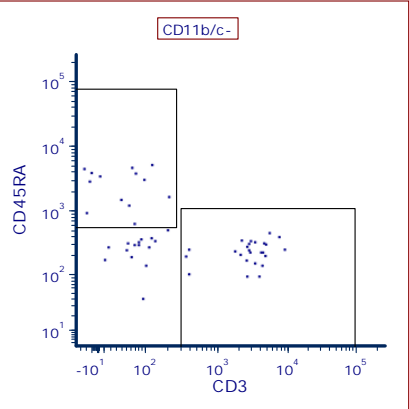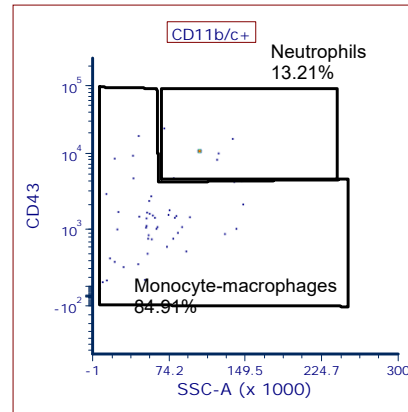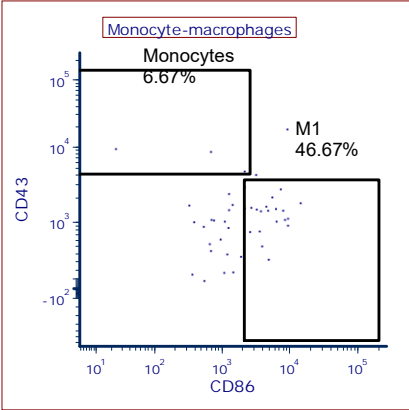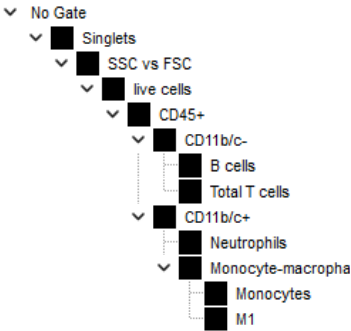

Supplement: Supplementary file 2 [file Image_1.pdf]
